# Dynamic fibroblast contractions attract remote macrophages in fibrillar collagen matrix

**DOI:** 10.1038/s41467-019-09709-6

**Published:** 2019-04-23

**Authors:** Pardis Pakshir, Moien Alizadehgiashi, Boaz Wong, Nuno Miranda Coelho, Xingyu Chen, Ze Gong, Vivek B. Shenoy, Christopher A. McCulloch, Boris Hinz

**Affiliations:** 1 Laboratory of Tissue Repair and Regeneration, Faculty of Dentistry, University of Toronto, Toronto, ON M5G 1G6 Canada; 2 Faculty of Dentistry, University of Toronto, Toronto, ON M5G 1G6 Canada; 3 Institute of Biomaterials and Biomedical Engineering, University of Toronto, Toronto, ON M5S 3G9 Canada; 4 Department of Chemistry, University of Toronto, Toronto, ON M5S 3E5 Canada; 5 Department of Physiology, University of Western Ontario, London, ON Canada; 6 Center for Engineering Mechanobiology and Department of Materials Science and Engineering, School of Engineering and Applied Science, University of Pennsylvania, Philadelphia, PA 19104 USA

**Keywords:** Motility, Cell migration

## Abstract

Macrophage (Mϕ)-fibroblast interactions coordinate tissue repair after injury whereas miscommunications can result in pathological healing and fibrosis. We show that contracting fibroblasts generate deformation fields in fibrillar collagen matrix that provide far-reaching physical cues for Mϕ. Within collagen deformation fields created by fibroblasts or actuated microneedles, Mϕ migrate towards the force source from several hundreds of micrometers away. The presence of a dynamic force source in the matrix is critical to initiate and direct Mϕ migration. In contrast, collagen condensation and fiber alignment resulting from fibroblast remodelling activities or chemotactic signals are neither required nor sufficient to guide Mϕ migration. Binding of α2β1 integrin and stretch-activated channels mediate Mϕ migration and mechanosensing in fibrillar collagen ECM. We propose that Mϕ mechanosense the velocity of local displacements of their substrate, allowing contractile fibroblasts to attract Mϕ over distances that exceed the range of chemotactic gradients.

## Abstract (web summary)

Macrophages play an important role in wound healing but the guidance cues driving macrophages to sites of repair are still not clear. Here the authors discover that macrophages are attracted to contracting fibroblasts by responding to locally sensed displacements of collagen fibres.

## Introduction

Macrophages (Mϕ) are first responders to organ injury and are crucial for repair, resolution, and re-establishing homeostasis of damaged tissue^1,2^. Stimulated by inflammatory cell infiltration, fibroblasts also populate the wound bed and are activated into myofibroblasts (MFs) that produce and contract collagenous extracellular matrix (ECM)^3^. Mϕ, continuously recruited from the circulation and local sources, thus share their environment with collagen-producing and -contracting cells. Coordinated activities of Mϕ and MFs support normal wound healing, but dysregulation of this process often results in excessive accumulation and contraction of ECM—a condition called fibrosis^1,3^. Stiff fibrotic ECM can severely impair the function of organs to the point of their failure. Scar stiffness also directly contributes to mechanical activation of various precursor cells into pro-fibrotic MFs, which occurs in synergy with pro-fibrotic cytokines such as transforming growth factor-β1 (TGF-β1)^3,4^. Mϕ are a prominent source of TGF-β1 in early wound repair and fibrosis but TGF-β1 only acts in very close range from its source and/or site of activation^5^. We and others have shown that proximity is crucial to allow crosstalk between Mϕ and contractile fibroblasts^5–7^, but it remains elusive how this proximity is established. Although directed Mϕ migration is being studied in the context of attraction to/of cancer cells^8^, it is not known whether fibroblasts and MFs are able to attract Mϕ and which factors are involved. Potential Mϕ-to-MF attraction mechanisms are paracrine signaling (chemotaxis) and/or ECM cues, such as strain, stiffness gradients, or topographies^9–11^. Physical cues have been shown to establish intercellular mechanical communication through the ECM to guide cell adhesion, migration, and differentiation^12,13^. Fibroblasts have been shown to apply forces to their substrate and create mechanical cues that can be sensed far beyond the signal source by cells sharing the same substrate^13,14^. The range of these mechanical signals can exceed that of chemotactic gradients (100–200 µm)^15^ and is enabled through fibrillar ECM, such as collagen and fibrin, which propagates small local deformations through the network^16,17^.

We demonstrate that local remodeling of collagen fibers by contracting MFs provides far-ranging mechanical cues in the ECM that attract and guide Mϕ over long distances. This guidance can occur independently from structural changes in the ECM and chemotaxis but requires an active source of ECM deformation. We propose that mechanical signals support Mϕ attraction to MFs during normal and dysregulated tissue repair where the secreted growth factor environment may be too complex to provide clear chemotactic clues.

## Results

### Fibroblasts produce large deformation fields in collagen

**Fig. 1:**
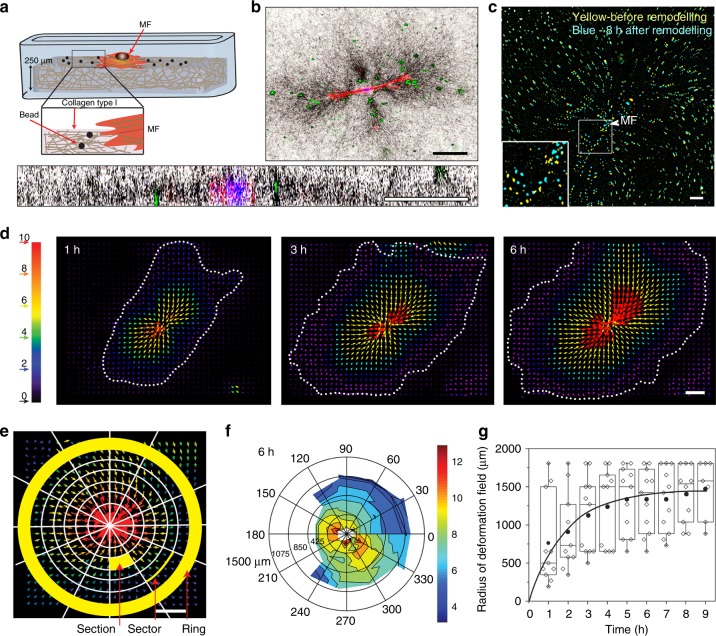
Myofibroblasts (`MFs) generate far reaching deformations in fibrillar collagen extracellular matrix (ECM). **a** Schematic of the experimental set-up. **b** Confocal reflection and fluorescence microscopy of the experimental set-up. Single MFs (F-actin, red) were attached to the top of fibrillar collagen ECM provided with surface marker beads (green) and allowed to remodel the ECM (reflection, black) for 3 h. Side view *z*-scanning (bottom) demonstrate the position of beads and MFs on the collagen surface. **c** MF contraction of ECM was quantified by analyzing position changes of marker beads over time, here shown in overlay before contraction (yellow) and after 8 h of ECM remodeling (blue) by MFs (position indicated by arrowhead). **d** Marker displacements were used to calculate MF-induced deformation fields using particle image velocimetry, displayed as vectors with color-coded magnitudes as indicated (red—high displacement). Deformation field boundaries were defined by displacement vector amplitudes ≤1 µm (dotted lines). **e** Breakdown of the deformation field into rings, sections, and sectors allowed computation of average vector magnitudes as a function of distance and direction from the contractile MF. **f** Such averages were used to align obtained deformation fields along their long axis and generating the overall average after 6 h remodeling (11 macrophages, 11 experiments) with color-coded magnitudes. **g** The radii of all MF-induced deformation fields were measured over time and medians are shown as center lines of box (25th to 75th percentiles) and whisker (minimum to maximum) plots; black circles and continuous are averages with exponential fit. All scale bars: 100 µm

Fibroblast contractions are sensed by other fibroblasts in the same fibrillar ECM^13^. To test whether mechanical communication through fibrillar ECM contributes to Mϕ attraction by contractile MFs, we first established low-density cell cultures (2 MFs cm^−2^) of MFs seeded onto three-dimensional (3D) collagen gels (2 mg ml^−1^, 250 µm thick) provided with surface bead position markers (Fig. 1a, b). MF-induced ECM deformation was calculated from bead displacements (Fig. 1c) using particle image velocimetry (PIV) for 0–8 h following MF attachment (Fig. 1d). The resulting heat-color-coded vector maps demonstrated maximum ECM deformation near MFs (on average 600 ± 100 µm standard deviation, red/yellow) and increasing diameters of ellipsoid deformation fields over time (Fig. 1d, Supplementary Movie 1). To average radii and local magnitudes of deformation fields for multiple MFs (*n* = 11, *N* = 11), all deformation fields were oriented along their short axis (*θ* = 0°) and sectors were defined according to distance and angle from the MF centroid (Fig. 1e). Plotting the average of the vector magnitudes per sector resulted in a non-uniformly shaped collagen deformation field created by bipolar-contracting MFs (Fig. 1f, 6 h). Analyzing average deformation field radii demonstrated that local deformations induced by MF contraction propagate far beyond the cell position in fibrillar collagen ECM (1300 ± 142 µm) with saturation reached after 6 h (Fig. 1g).

### Mϕ actively migrate toward MFs on collagen ECM

**Fig. 2:**
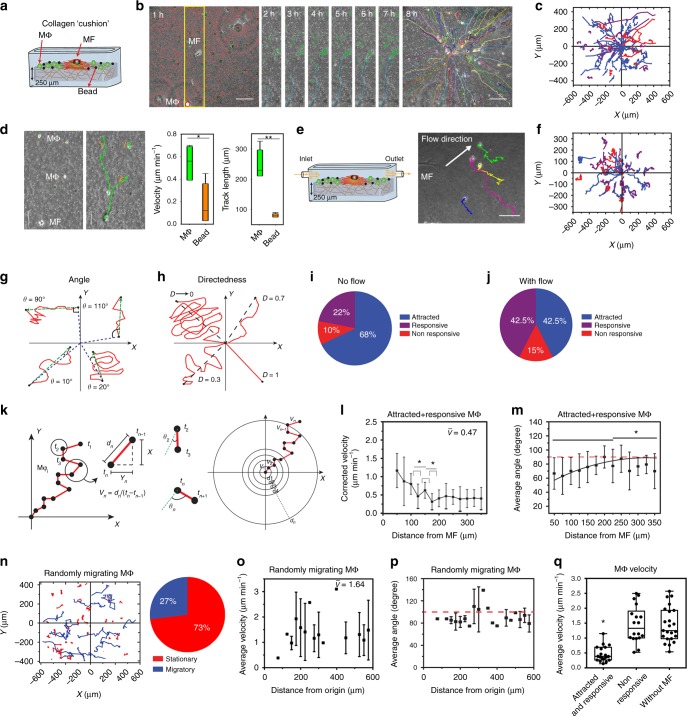
Macrophages (Mϕ) are attracted to myofibroblast (MF) on fibrillar collagen extracellular matrix (ECM). **a** Mϕ were co-cultured with MFs on top of fibrillar collagen ECM with surface marker beads and **b** recorded for 8 h at one frame per 5 min. Mϕ (green colored) and beads (red colored) were automatically tracked in phase-contrast videos. **c** Mϕ tracks (blue-attracted Mϕ, purple-responsive Mϕ, red-non-responsive Mϕ) were plotted with respect to the MF position (0,0). **d** Mϕ track lengths and velocity were compared with displacement of adjacent microbeads in box (25th to 75th percentiles) and whisker (minimum to maximum) plots with center lines showing medians (20 experiments with average 5 image fields, *p ≤ 0.05 and **p ≤ 0.01, Student’s *t* test). All Mϕ tracks were subsequently corrected for passive bead dragging. **e** Fluid flow was applied to eliminate possible chemotactic gradients and **f** Mϕ tracks were analyzed. **g** Total migration angle (*θ*) was defined as the angle between the line connecting the starting point of the Mϕ trajectory and the MF centroid and the line connecting the first and last point of the Mϕ trajectory. **h** Directedness (*D*) was defined by the quotient of Euclidian distance and track length. Pie charts summarize Mϕ migration behavior in **i** no flow conditions (145 Mϕ, 20 experiments) and **j** under flow (87 Mϕ, 12 experiments). **k** Local migration velocity (traveled distance *d*_n_ between two image frames) and migration angle *θ*_n_ to the MF position were calculated for every frame pair. **l** Local velocities and **m** angle changes of attracted and responsive Mϕ were plotted over distance to the MF (93 Mϕ, 20 experiments). **n** Mϕ trajectories were analyzed without MFs present (random migration). **o** Local migration velocities and **p** angles of Mϕ migration with respect to the image center (origin) (82 Mϕ, 10 experiments). Shown are medians ± value ranges (*p ≤ 0.05 using analysis of variance (ANOVA) followed by post hoc Tukey’s multiple comparison test). Slope changes were considered significant at *p ≤ 0.05, using slope comparison test. **q** Average local migration velocities were compared between attracted and responsive Mϕ (*n* = 19), non-responsive Mϕ (*n* = 18), and Mϕ migrating randomly without MFs (*n* = 23). Shown are medians ± value ranges (*p ≤ 0.05 using ANOVA followed by post hoc Tukey’s multiple comparison test)

To test whether MF-generated ECM deformations are detected by Mϕ, we added Mϕ (ratio 10:1) to MFs on collagen ECM after 1 h, followed by videomicroscopy for an additional 8 h (Fig. 2a, b). Tracking Mϕ relative to the position of MFs demonstrated directed Mϕ migration toward MFs (Fig. 2c, Supplementary Movie 2). Active Mϕ migration was 2.7-times faster and more persistent than passive dragging of adjacent marker beads with the ECM (Fig. 2d, Supplementary Movie 3). In all subsequent experiments, Mϕ velocity was corrected for passive dragging to assess of Mϕ attraction by active migration only. Mϕ attraction was independent from putative MF chemotactic factors because elimination of chemotactic gradients by applying slow fluid flow (low shear force of 0.5 N m^−2^) did not significantly affect Mϕ migration behavior (Fig. 2e, f, Supplementary Movie 4).

Next, we analyzed all Mϕ migration trajectories over 8 h for total migration angle *θ*, i.e., the angle between the lines connecting Mϕ starting and end point (net displacement) and between Mϕ starting point and MF centroid (Fig. 2g). Directedness (*D*) was defined as net displacement divided by total travel path length (Fig. 2h). Using these unbiased descriptors, we classified Mϕ migration behavior into three categories: (1) Non-responsive Mϕ that either did not migrate (travel path length ~0 µm) or did not exhibit MF-directed behavior (*D* ≤ 0.3 or *θ* ≥ 90°). (2) Attracted Mϕ that clearly migrated toward MFs (*θ* < 90° and *D* > 0.5), and (3) Responsive Mϕ that only weakly fulfilled the directed migration criteria (*θ* > 90° and 0.3 > *D* ≤ 0.5). On collagen ECM, 68% Mϕ were attracted, 22% were responsive, and 10% non-responsive to MFs (Fig. 2i, *n* = 145, *N* = 20). In the presence of fluid flow and thus absence of chemotactic gradients, 42% Mϕ were attracted, 42% responsive, and 15% non-responsive (Fig. 2j, *n* = 87, *N* = 12). As an additional control to exclude chemotaxis, Mϕ were co-cultured with MFs on tissue culture plastic (TCP) coated with 5-µm-thin monomeric and fibrillar collagen (Supplementary Fig. 1). On both non-deformable ECMs, the percentage of attracted Mϕ was 5.5-fold lower than on thick, deformable fibrillar collagen (Supplementary Fig. 1).

To determine the efficacy of Mϕ to detect MFs as a function of their distance, we analyzed the tracks of all attracted Mϕ for corrected velocity (*V*_n_) and angle changes (*θ*_n_) between frame acquisitions (5 min) (Fig. 2k). Mϕ velocity increased (Fig. 2l) and migration angle decreased (Fig. 2m) with decreasing distance to MFs. Although Mϕ were attracted over distances as far as 600 µm (Fig. 2c–f, Supplementary Movie 1), velocity and directedness of Mϕ migration were highest within a radius of 200 ± 50 µm around the MF (Fig. 2m). Control experiments with Mϕ on collagen without MFs validated the analysis (Fig. 2n); all Mϕ fulfilled the non-responsive criteria (*D* ≤ 0.3) with 73% being stationary (*D* ~ 0) and 27% migrating randomly (*D* ≤ 0.3) with an average speed of 1.64 µm min^−1^ (Fig. 2o, p) (*n* = 82, *N* = 10). Notably, responsive and attracted Mϕ migrated with three-fold lower average local velocity (*v* = 0.47 ± 0.26 µm min^−1^) than non-responsive Mϕ (*v* = 1.45 ± 0.61 µm min^−1^) or randomly migrating Mϕ-only controls (*v* = 1.44 ± 0.57 µm min^−1^) (Fig. 2q). The molecular basis for the decreased velocity of directedly vs. randomly migrating Mϕ is yet unclear.

**Fig. 3:**
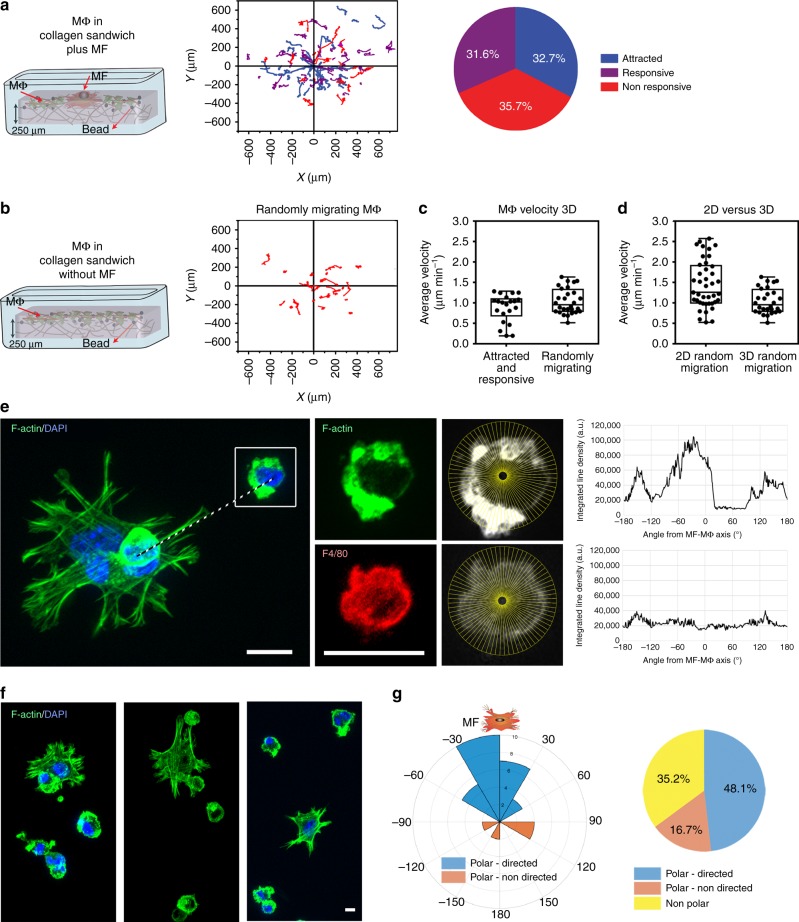
Macrophages (Mϕ) are attracted and orient toward myofibroblasts (MFs) within fibrillar collagen extracellular matrix (ECM). Mϕ within three-dimensional (3D) collagen gel sandwich cultures were tracked over 12 h either **a** with or **b** without MFs being present. Mϕ tracks (blue-attracted Mϕ, purple-responsive Mϕ, red-non-responsive Mϕ) were plotted with respect to the MF position or the image center (0,0) (**a**, 40 Mϕ, 8 experiments; **b**, 28 Mϕ, 8 experiments). Average local migration velocities were compared between **c** Mϕ that were attracted and responsive (*n* = 22) or Mϕ that were non-responsive (*n* = 28) and **d** between Mϕ randomly migrating in 2D (*n* = 41) or 3D collagen (*n* = 28). Shown are medians (center lines) of box (25th to 75th percentiles) and whisker (minimum to maximum) plots. **e** Mϕ and MFs were co-cultured within 3D collagen gel sandwich cultures for 3 h, followed by staining for F-actin (Phalloidin, green), Mϕ marker (F4/80, red) and nuclei (DAPI, blue). Integrated F-actin fluorescence intensity profiles along lines drawn from the Mϕ centroid in 1° angle distances (shown for every 5°) over 360° were used to identify the leading (highest actin content). Zero angle was defined by a line connecting Mϕ and MF centroids. **f** Three additional examples for Mϕ orientations toward MFs in the same ECM. **g** Peak angles from **e** were summarized to relate angle orientation of Mϕ to the position of MFs (0°) in rose plots. Mϕ with orientation angles <60° were considered to have polar-directed orientation toward MFs (*n* = 57). All scale bars: 25 µm

Tracking non-restricted Mϕ migration on top of collagen gels allowed fast and non-invasive quantification of Mϕ responses to contracting MFs. However, Mϕ in vivo migrate in 3D tissue environments. To validate that Mϕ mechanosensing of MF contractions also occurs in 3D, both cell types were sandwiched between two layers of 3D collagen (Fig. 3a) with Mϕ only as control (Fig. 3b) and tracked. The percentage of attracted Mϕ was reduced by ~2-fold in 3D (Fig. 3a, 32.7%) vs. two-dimensional (2D) (Fig. 2i, 68%) with moderately higher fractions of responsive Mϕ (~1.4-fold) in 3D (31.6%) vs. 2D (22%). The attraction and directiveness criteria (Fig. 2g, h) depend on Mϕ migration distances between acquisition frames (=velocity). Because average Mϕ velocities were ~1.4-fold lower in 3D (*v* = 1.04 ± 0.31 µm min^−1^) vs. 2D (*v* = 1.44 ± 0.57 µm min^−1^) (Fig. 3c, d), we used F-actin accumulation at the leading cell edge as additional measure for Mϕ polar directionality toward MFs in the 3D environment (Fig. 3e). After 3 h co-culture, responding Mϕ oriented their F-actin toward MFs with trailing nuclei (Fig. 3e, f). Mϕ were considered polar-directed at angles of <60° between the Mϕ polar axis and the connection of Mϕ and MF centroids (Fig. 3g, 48.1%). At larger angles, Mϕ were classified as polar but non-directed (16.7%), and Mϕ with no obvious orientation were considered non-polar (35.2%) (Fig. 3g). Hence, although the proportions of Mϕ responding to MFs were high within 3D collagen (64.3% from migration and 48.1% from orientation), Mϕ migration analysis on top of collagen gel cultures was more robust and largely independent from possible steric effects of 3D ECM in different remodeling states. All subsequent experiments were thus performed with cells cultured on top of 3D collagens.

### ECM structural cues are insufficient to attract Mϕ to MFs

**Fig. 4:**
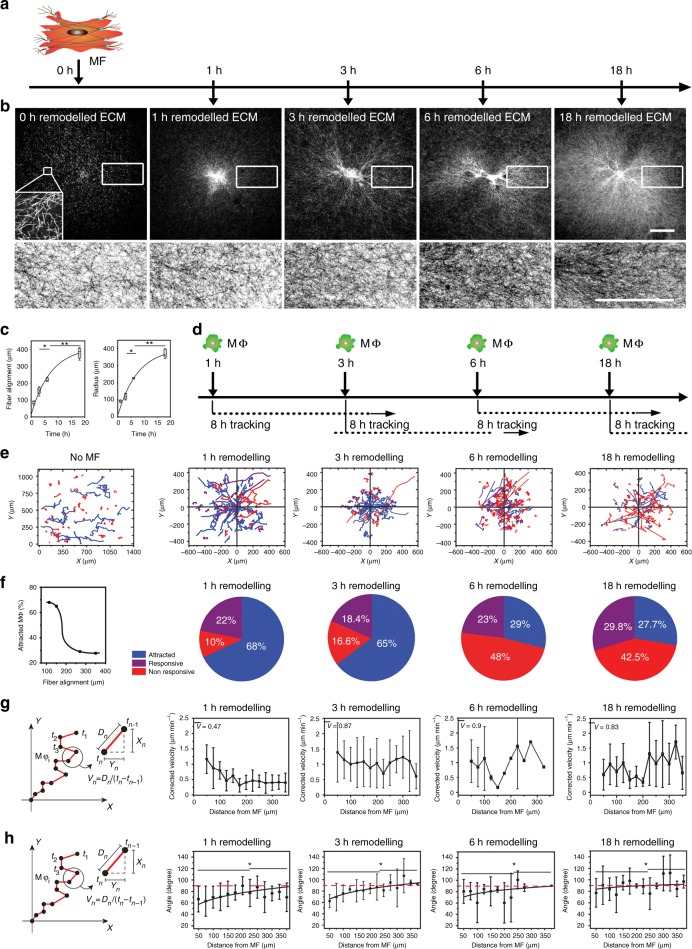
Structural cues in collagen networks do not enhance macrophage (Mϕ) migration to myofibroblasts (MFs). **a** MFs remodeled collagen extracellular matrix (ECM) for 1–18 h. **b** Confocal reflection microscopic images were taken of collagen ECM, remodeled by MF (in image center) after 1, 3, 6, and 18 h; higher magnifications of the boxed regions are shown inverted to highlight fibrillar collagen. **c** Collagen fiber alignment and radius of the structural changes in the ECM were calculated and plotted over time of MF remodeling. Data points are averages ± SD (**p ≤ 0.01 using analysis of variance followed by post hoc Tukey’s multiple comparison test). **d** MFs were allowed to remodel collagen ECM for 1–18 h prior to adding Mϕ that were then tracked for another 8 h. **e** Trajectories of migratory Mϕ are plotted with respect of the MF position as a function of pre-remodeling time. **f** Percentages of attracted, responsive, and non-responsive Mϕ are shown for the different ECM remodeling states (pie charts) and the percentage of attracted Mϕ was plotted as a function of collagen fiber alignment calculated from confocal reflectance images. **g** Local Mϕ migration velocities, corrected for ECM dragging, were plotted as a function of distance to the MF for every pre-remodeling condition. **h** Migration angles were plotted as a function of distance to the MF. Data points are medians with error bars representing minima and maxima (93 Mϕ, 20 independent experiments). Slope changes were considered significant at *p ≤ 0.05, using slope comparison test. All scale bars: 100 µm

Previous studies have shown that monocytes can detect structural ECM cues, such as topographies, fibril alignments, and stiffness gradients^18^. To test the role of ECM structural changes, MFs were allowed to induce clear structural changes in the fibrous collagen network over 1–18 h of pre-remodeling, as shown by confocal reflection microscopy (Fig. 4a, b). Collagen fibril alignment and radius of the remodeling field were maximal after 18 h (Fig. 4c). Mϕ were then introduced to differently pre-remodeled ECM with continued presence of MFs and migration was tracked for another 8 h (Fig. 4d). The percentage of attracted Mϕ decreased with increasing degree of ECM remodeling and collagen alignment by the MF (Fig. 4e, f, 2.6-fold between 1 h and 18 h). Mϕ directional migration toward MFs was most efficient on 1–3 h MF-remodeled collagen ECM, where Mϕ velocities increased and angles decreased with proximity to MFs in a 200 ± 50 µm perimeter (Fig. 4g, h). However, average Mϕ velocities $\overline {({\mathrm{V}})}$ over all distances were higher on 3–18 h remodeled ECM compared with 1 h of remodeling (Fig. 4g). Quantifying the numbers of F4/80-positive Mϕ accumulating in a radius of 400 µm around MFs confirmed higher Mϕ-to-MF attraction in ECM with lower remodeling degrees (Supplementary Fig. 2). Collectively, these results show that pre-alignment of collagen fibers does not enhance direction sensing of Mϕ toward contractile MFs. Hence, structural ECM cues are neither required nor sufficient to promote Mϕ-to-MF attraction.

### Mϕ follow dynamic pulling events transmitted through ECM

**Fig. 5:**
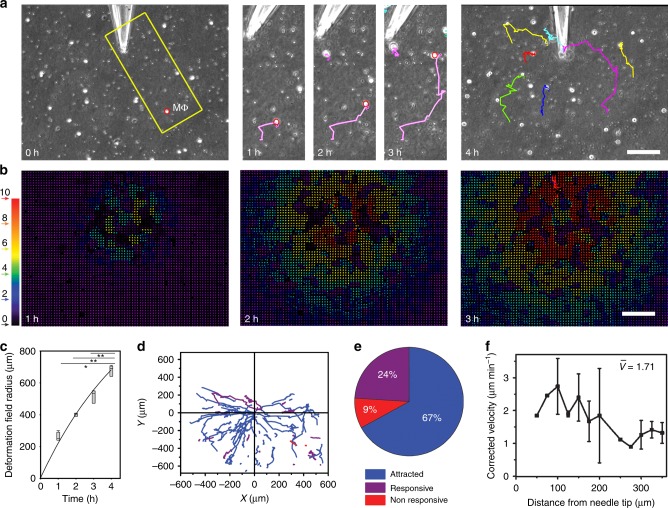
Macrophages (Mϕ) are attracted by local pulling events in collagen extracellular matrix (ECM). **a** Mϕ were seeded onto collagen ECM with surface marker beads without myofibroblast (MF) but with microneedles inserted 5 µm into the 200-µm-thick collagen gel. Lateral collagen deformation, mimicking MF contraction, was achieved by sucking collagen fibers into the tip using negative pressure. Mϕ migration was tracked from phase contrast movies. **b** Color-coded vector maps were obtained from particle image velocimetry (PIV) of bead displacements to represent the induced deformation fields as indicated (red—high displacement). **c** Deformation field radii of microneedle-induced deformation fields are plotted over time and shown as medians (center lines) of box (25th to 75th percentiles) and whisker (minimum to maximum) plots (*p < 0.05 and **p ≤ 0.01 using analysis of variance followed by post-hoc Tukey’s multiple comparison test). **d** Mϕ trajectories are plotted over 4 h with respect to the position of the microneedle (0,0) and **e** classified into attracted, responsive, and non-responsive Mϕ. **f** Median values of the dragging-corrected velocities of attracted Mϕ are plotted as a function of distances to the microneedle tip. Error bars represent the minima and maxima, 111 Mϕ, 12 independent experiments. Only experiments were considered for analysis where collagen created a tight seal with the tip and suction did not induce any flow, as controlled by fluorescent particles added to the culture medium. All scale bars: 100 µm

The fact that collagen fibril alignment was not conducive and even counterproductive for Mϕ attraction raises the question of the mechanical signal produced by MFs and sensed by distant Mϕ. We have previously shown that MFs cultured in 2D and 3D environments exhibit periodic contractile events^19,20^. To test whether dynamic pulling serves as Mϕ attraction signals, incremental collagen ECM deformations were induced using micromanipulator-controlled microneedles (Fig. 5a, Supplementary Movie 5) with frequency and amplitude measured for MFs^19^. Like MFs (Fig. 1), microneedle pulling created graded deformation fields with higher ECM displacements in tip vicinity (Fig. 5b). The time to reach a deformation field radius of 600 µm (4 h) was accelerated 1.9-fold compared with MF-created deformation fields (Fig. 5c). Because microneedle pulling was not feedback-controlled by ECM resistance, as opposed to contracting MFs, deformation field growth did not saturate within this time (Fig. 5c). Mϕ responded to the induced mechanical signal by migrating to the microneedle tip (Fig. 5a–c). Consistent with migration toward contracting MFs, 67% of the tracked Mϕ were attracted, 24% responsive, and 9% did not respond to 4 h of ECM pulling by the microneedle (Fig. 5d, e). Corrected Mϕ velocities increased with decreasing distance to the source of ECM pulling (Fig. 5f). These results identified the presence of a dynamic force source as the critical signal initiating and directing Mϕ migration in fibrillar ECM.

**Fig. 6:**
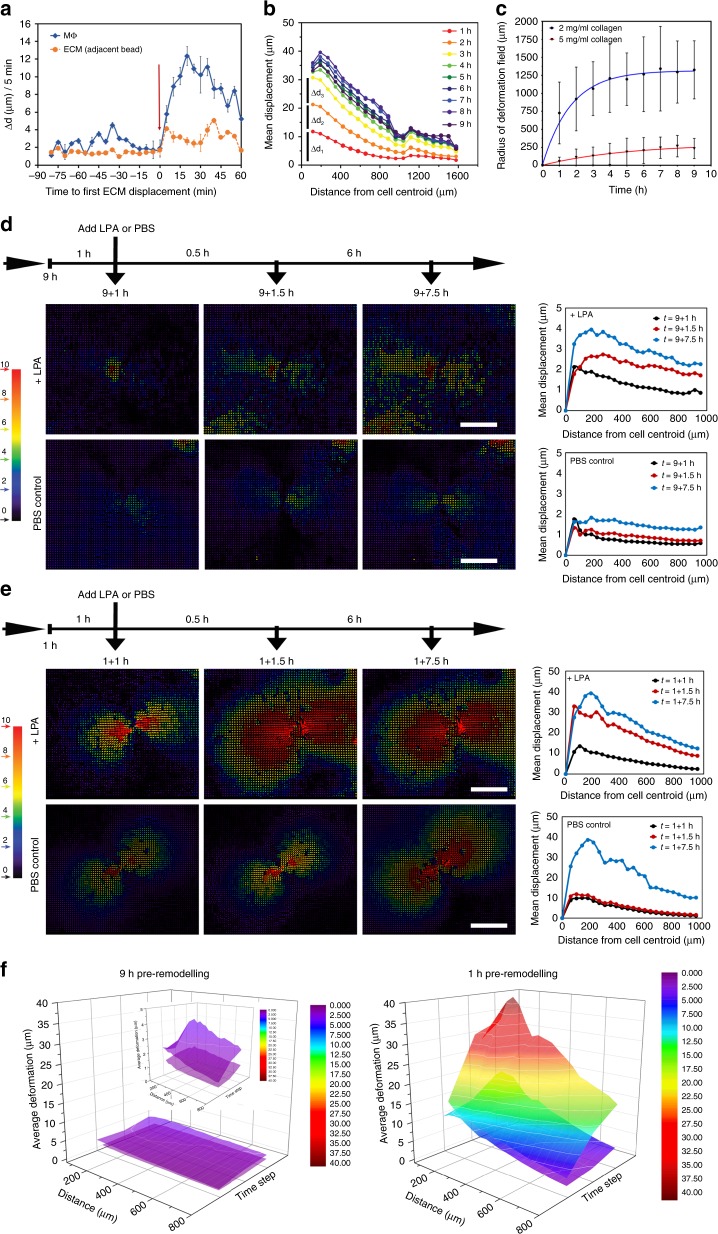
Macrophages (Mϕ) detect and respond local displacements in their extracellular matrix (ECM) exceeding a threshold magnitude. **a** To determine the displacement amplitude change Δ*d* required to trigger an Mϕ response, Mϕ displacements were co-analyzed with their immediate ECM substrate (marker beads). Only attracted Mϕ with a clear change in behavior from undirected to biased movement toward the myofibroblast (MF) were included in this detailed analysis (10 Mϕ, 6 independent experiments). Combined Δ*d*_Mϕ_ and Δ*d*_ECM_ data were aligned and plotted with respect to the first detectable acceleration in bead/ECM displacement (arrow, *t* = 0 min). Shown are averages ± SD. **b** Average ECM displacement magnitudes were plotted over 1–8 h of MF contraction (Fig. 1f) and analyzed for amplitude changes between time points (Δ*d*_n_) at any given distance from the MF. **c** The radii of MF-induced deformation fields were measured over time on either 2 mg ml^−1^ (blue) or 5 mg ml^−1^ (red) collagen gels; averages are shown ±SD (*n* = 5). **d** Marker displacements in collagen gels were used to calculate MF-induced deformation fields that are displayed in color-coded vector diagrams with MF position in the center as indicated (red—high displacement). MFs were allowed to pre-remodel the ECM for 8 h (reference point for particle image velocimetric calculations). Deformation was recorded after another 1 h (9+1 h) without treatment and 1.5 and 7.5 h after adding lysophosphatidic acid (10 ng ml^−1^) or phosphate-buffered saline control as indicated. Scale bars: 500 µm. Graphs show average ECM displacement magnitudes over distance from the MF centroid (*N* = 3). **e** Same experimental set-up as in **d** but after 1 h pre-remodeling. **f** Summary three-dimensional plots of the data shown in **d**, **e**

Since Mϕ were most efficiently attracted to MFs within the first 3 h of collagen remodeling when the deformation field radius changed most significantly (Fig. 1d–g), we hypothesized that Mϕ respond to changes in the amplitude of collagen displacements Δ*d* over time. Displacements of Mϕ (Δ*d*_Mϕ_, not corrected for ECM displacement) were co-analyzed with their immediate ECM substrate (Δ*d*_ECM_, adjacent marker bead displacement) in MF direction (Fig. 6a). Only attracted Mϕ with a clear change in behavior from undirected to biased movement toward the MF were included in this detailed analysis (*n* = 10). Combined Δ*d*_Mϕ_ and Δ*d*_ECM_ data were aligned and plotted with respect to the first detectable acceleration in bead/ECM displacement (Fig. 5a, arrow, *t* = 0 min). This alignment allowed to uncouple Mϕ behavior from their position relative to the MF and time and thus analyzing active Mϕ migration exclusively as a function of local ECM displacement. At Δ*d*_ECM_ ≤ 2 µm, Mϕ were dragged with their local ECM at the same speed of 0.82 ± 0.36 µm min^−1^ (2 µm per 5 min) and did not exhibit active migration. In contrast, ECM displacement amplitudes of Δ*d*_ECM_ ≥ 4 µm triggered active Mϕ migration toward the MF, i.e., Mϕ moved faster than their associated bead/ECM. The switch from passive to active Mϕ displacement occurred within 5 min between acquisition frames (Fig. 6a). Thus Mϕ were capable of sensing contractile MF by detecting local displacements in their ECM substrate over time that needed to surpass a strain velocity of ≥0.8 µm min^−1^ in our experimental conditions.

### MF remodeling reaches tensional homeostasis

To understand how increased ECM remodeling results in decreased attraction of Mϕ to MF, we analyzed the average ECM displacement magnitudes (Fig. 1e, analyzed by ring) over 1–8 h of MF contraction. Amplitude changes between time points (Δ*d*_n_) were largest up to 3–4 h of remodeling at any distance to the MF. Between 4 and 9 h, all Δ*d* became negligible, i.e., deformation fields were stable at these time points (Fig. 6b). Remodeling by MFs gradually strains and thereby increases the mechanical resistance of collagen networks, as appreciated from the high degree of collagen fiber alignment after 6–9 h of remodeling (Fig. 4a–c). We propose that, at this stage, collagen network resistance and fibroblast forces reach tensional homeostasis, analogous to a person straining a sport expander as much as the strength of the individual allows to maintain isometric force. Consistently, MFs produce 5.6-fold smaller deformation fields in denser collagen gels with higher mechanical resistance (5 mg ml^−1^) compared to loser gels (2 mg ml^−1^) (Fig. 6c).

To demonstrate that MFs reach tensional homeostasis as opposed to stop contracting, contraction was acutely enhanced using lysophosphatidic acid (LPA)^21^ after MFs had established stable deformation fields in 2 mg ml^−1^ collagen gels after 9 h (Fig. 6d) or early during remodeling for 1 h (Fig. 6e). Resulting changes in ECM displacement were quantified with respect to the status before addition of LPA and plotted as a function of distance from the MF centroid. LPA-induced MF contraction resulted in moderate additional displacement of the 9 h pre-remodeled ECM with uniform amplitudes within a radius of 100–1000 µm around the MF (Fig. 6d–f, LPA, 9+1.5 h). Deformation of 9 h pre-remodeled collagen in unstimulated controls was negligible (Fig. 6d–f, phosphate-buffered saline (PBS) control). Inducing MF contraction before reaching tensional homeostasis (1 h pre-remodeling) resulted in 10-times larger collagen displacements compared to induction after 9 h (Fig. 6e, f, 1+1 h). Displacements were the largest close to the MF and decreased with distance (Fig. 6e, f, 1+1.5 h). Without LPA stimulation (Fig. 6e, f, 1 h, PBS control), collagen remodeling characteristics followed the course described before (Fig. 6b). These experiments demonstrated that MF maintain isometric contraction after reaching tensional homeostasis in organized collagen gels. At tensional homeostasis, bursts of MF contraction force are transmitted throughout the network with little loss in deformation amplitude over distance.

### Mϕ attachment and mechanosensing mechanisms

**Fig. 7:**
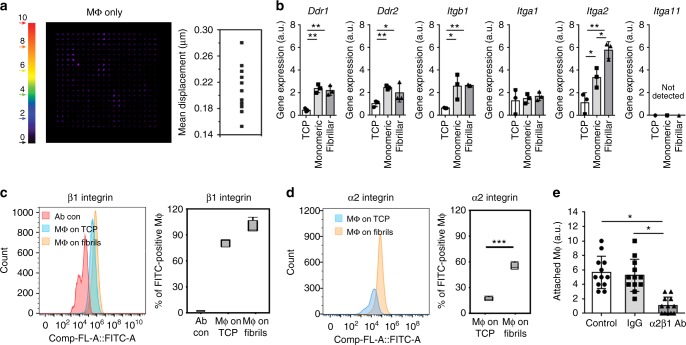
Macrophage (Mϕ) traction on fibrillar collagen extracellular matrix. **a** Marker displacements were used to calculate Mϕ-induced deformation fields using particle image velocimetry, displayed as vectors with color-coded magnitudes and average vector magnitudes per Mϕ are summarized in a graph (15 Mϕ, 11 independent experiments). **b** Quantitative reverse transcription polymerase chain reaction assessment of collagen I receptors in Mϕ grown for 7 days on uncoated tissue culture plastic (TCP) or TCP coated with monomeric and fibrillar collagen. Gene expression values were normalized to the average expression of house-keeping genes *G6pd*, *Gapdh*, and *Hmbs* (*N* = 3). **c**, **d** For flow cytometry, primary mouse Mϕ were cultured on fibrillar collagen-coated surfaces for 6 h, detached, and live cells stained for **c** active β1 integrin-detecting antibody (CD29, clone 9EG7), and **d** α2β1 integrin (CD49b) fluorescein isothiocyanate-coupled antibody followed by secondary fluorescent antibody. Mϕ were incubated with secondary antibody only for control. Percentages of β1 and α2 integrin-positive Mϕ are shown as medians (center lines) of box (25th to 75th percentiles) and whisker (minimum to maximum) plots (*N* = 3). **e** Suspended Mϕ were seeded onto fibrillar collagen-coated substrates in the presence of α2β1 integrin blocking or control antibodies and percentages of attached Mϕ were quantified after 1 h (*N* = 3). Graphs show averages ± SD, (*p < 0.05, **p ≤ 0.01, ***p < 0.001 using analysis of variance followed by post hoc Tukey’s multiple comparison test)

To mechanosense and mechanotransduce ECM displacements into directed migration, Mϕ need to engage with collagen. As a first possible indication of this engagement, directed migration of Mϕ on 2D substrates was three-fold lower than that of randomly migrating Mϕ (Fig. 2q). Furthermore, although being orders of magnitude smaller than what was generated by MFs, Mϕ produced tractions on fibrillar collagen substrates during migration that generated ECM displacements of ≤0.28 µm (Fig. 7a). To identify potential Mϕ mechanoreceptors, we first assessed the expression of known fibrillar collagen I-binding receptors discoidin receptors (DDRs) DDR1 (*Ddr1*) and DDR2 (*Ddr2*) and the subunits of integrins α1β1 (*Itga1*, *Itgb1*), α2β1 (*Itga2*), and α11β1 (*Itga11*) by quantitative reverse transcription polymerase chain reaction (qRT-PCR). Expression of *Ddr1*, *Ddr2*, *Itgb1*, and *Itga2* but not *Itga2* was higher in Mϕ grown on monomeric collagen I, compared to growth on TCP; *Itga11* was not detected (Fig. 7b). Of all the receptors, only the α2 integrin subunit was significantly higher expressed in Mϕ on fibrillar collagen I compared to monomeric collagen-coated surfaces (Fig. 7b). At similar β1 integrin expression levels (Fig. 7c), integrin α2 protein was also four-fold upregulated on the surface of Mϕ cultured on fibrillar collagen-coated ECM compared to Mϕ cultured on TCP (Fig. 7d). Further, incubation of suspended Mϕ with function-blocking α2β1 integrin antibodies resulted in five-fold reduced adhesion to fibrillar collagen (Fig. 7e).

**Fig. 8:**
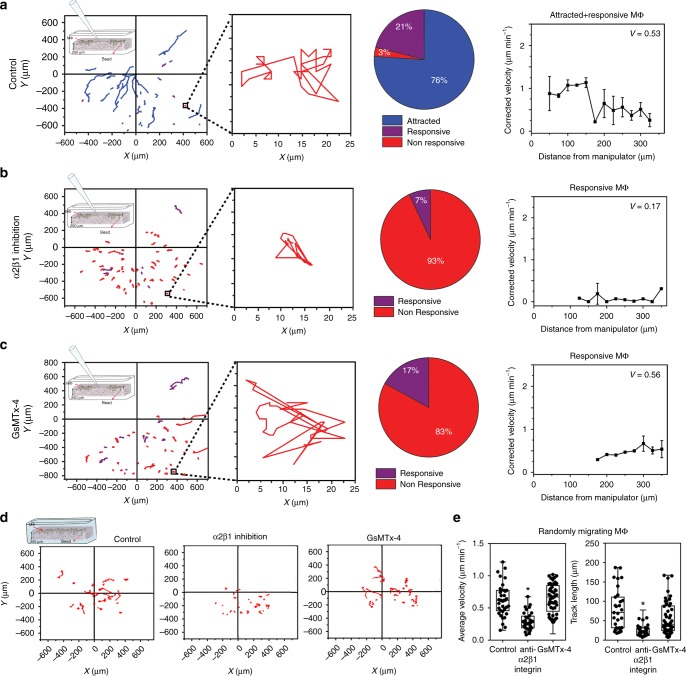
Macrophage (Mϕ) mechanosensation mechanisms on fibrillar collagen extracellular matrix (ECM). **a** Mϕ were seeded onto 250-µm-thick collagen ECM with surface marker beads and overlaid with a 20-µm-thin film of polymerized collagen before inserting a microneedle 5 µm into the 270-µm-thick collagen gel. Lateral collagen deformation, mimicking myofibroblast contraction, was achieved by sucking collagen fibers into the tip using negative pressure. Mϕ migration was tracked from phase-contrast movies and tracks are plotted with respect to the position of the microneedle (0,0). Individual representative tacks are zoomed to discriminate between local migration and immobility. Mϕ migration is classified into attracted, responsive, and non-responsive Mϕ without treatment in pie charts (44 Mϕ, 3 experiments). Median values of corrected velocities of attracted Mϕ are plotted as a function of distances to the microneedle tip with error bars representing minima and maxima. **b** Same experimental set-up and analysis as in **a** but in the presence of α2β1 integrin blocking antibody (10 µg ml^−1^) (60 Mϕ, 3 experiments). **c** Same experimental set-up and analysis as in **a** but in the presence of stretch-sensitive ion channel blocker GsMTx-4 (3 µM) (46 Mϕ, 3 experiments). **d** Same experimental set-up and analysis as in **a**–**c** but without micromanipulator. **e** Average local migration velocities and track lengths of randomly migrating (no stimulus) Mϕ are compared between control (*n* = 39), α2β1 integrin inhibition (*n* = 31), and GsMTx-4 (*n* = 53). Shown are medians ± value ranges (*p ≤ 0.05 using analysis of variance followed by post-hoc Tukey’s multiple comparison test)

To evaluate the role of α2β1 integrin in Mϕ mechanosensation and prevent detachment, Mϕ were cultured for 1 h in collagen sandwich cultures (Fig. 3) and actuated microneedles were used as mechanical stimulus (Fig. 5). Without drug treatment, 76% of Mϕ (*n* = 44) were attracted, 21% responsive, and 3% did not respond to ECM pulling by the microneedle within 4 h (Fig. 8a). Inhibition of α2β1 integrin almost completely abolished Mϕ migration on fibrillar collagen ECM and attraction to the contraction source (Fig. 8b). Collectively, these results identify α2β1 integrin as main fibrillar collagen receptor in cultured mouse Mϕ that is required for Mϕ migration on/in collagen. Adhesion-dependent cell migration allows for mechanosensing by stretch-dependent ion channels^22^. Indeed, adding the stress-sensitive ion channel blocker GsMTx4^23^ resulted in impaired directional migration of Mϕ toward actuated microneedles (Fig. 8c). The velocity of GsMTx4-treated responsive Mϕ was like that of non-treated Mϕ, showing that the drug did not interfere with random migration (Fig. 8a–d). Moreover, GsMTx4 treatment did not alter average track lengths (Fig. 8c–e, 57.35 ± 41.84 µm) and velocity (Fig. 8e, *v* = 0.64 ± 0.23 µm min^−1^) of randomly migrating Mϕ in the absence of a contraction source, compared with untreated controls (79.11 ± 51.27 µm and *v* = 0.63 ± 0.24 µm min^−1^). In contrast, inhibition of α2β1 integrin significantly reduced track lengths (Fig. 8b–e, 24.41 ± 16.05 µm) and velocity (Fig. 8e, *v* = 0.29 ± 0.13 µm min^−1^). Hence, Mϕ mechanosensing in collagen ECM is dependent on stretch-sensitive membrane ion channels. It is conceivable that α2β1 integrin is also involved in Mϕ mechanosensing, which however, could not be uncoupled from its adhesion function required for Mϕ migration on fibrillar collagen.

### Computational model of Mϕ-to-MF mechanical attraction

**Fig. 9:**
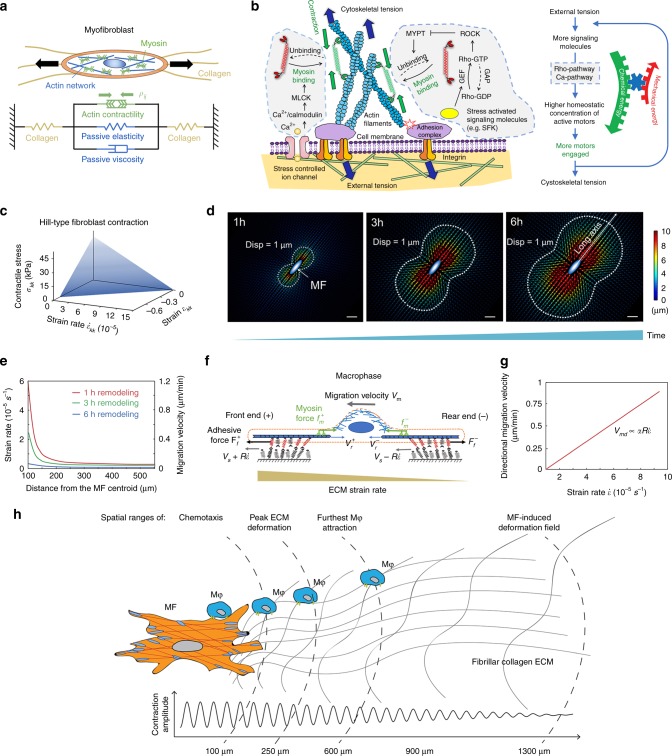
Multi-scale computational model of myofibroblast (MF) attraction by MF-induced collagen contractions. **a** Cellular-level model to obtain the spatiotemporal distribution of deformation fields created by MFs. **b** Schematic description of the cell contractility model. The Rho-ROCK and Ca^2+^ pathways are regulated by the stresses from the extracellular matrix (ECM). When the cell is under tension, a series of biochemical process is activated, and ultimately, more myosin motors switch from inactive state (red) to active state (green) binding to the cytoskeleton, creating more force dipoles in the cell. Collectively, these force dipoles lead to contraction of the ECM. **c** The mechanochemical model adopts a Hill-type contractile behavior for the myofibroblasts. **d** The predicted spatiotemporal variation of displacement fields in collagen. Scale bar: 100 μm. **e** Strain rate and corresponding macrophage (Mϕ) migration speed fields of the collagen substrate along the cell long axis shown in **d** as a function of distance. **f** Molecular-level model to determine the migration speed of the Mϕ. **g** The predicted linear relation between substrate strain rate and Mϕ migration speed $V_{{\mathrm{md}}} = \alpha R{\dot{ \epsilon }}$. **h** The working model schematizes the attraction range of Mϕ in the experimental system in relation to literature values reporting chemotaxis ranges in similar open culture systems

Next, we developed a multi-scale computational model to elucidate how spatiotemporal variations of strains induced by MFs can result in Mϕ responses. In the model, MFs are rigidly anchored to the ECM through focal adhesions and contain active contractile myosin motors and passive crosslinkers (e.g., α-actinin) whose binding and unbinding characterize the viscoelastic behavior of the F-actin cytoskeleton (Fig. 9a, Supplementary Fig. 3, Supplementary Note 1)^24,25^. The active component *ρ*_*ij*_ characterizes contractile force generated by myosin motors whose binding to the cytoskeleton is influenced by time-varying stress and strain through mechanosensitive pathways (e.g., Rho-ROCK, Ca^2+^) (Supplementary Eq. 6, Fig. 9b). Overall, the MF model behaves similar to muscle governed by the Hill relation^25^ (Fig. 9c), which relates cell volumetric contractile stress *σ*_*kk*_ with the speed of volumetric contraction ${\dot{ \epsilon }}_{kk}$ by

1 $$\frac{{\sigma _{kk}}}{{\sigma _{\mathrm{m}}}} - \frac{{{\dot{ \epsilon }}_{kk}}}{{{\dot{ \epsilon }}_{\mathrm{m}}}} = 1$$

where *σ*_m_ can be identified as the stall stress, and ${\dot{ \epsilon }}_{\mathrm{m}}$ is the maximum rate of contraction (Supplementary Note 1). Contraction speed ${\dot{ \epsilon }}_{kk}$ decreases with the increasing stress *σ*_*kk*_ in Eq. (1). Collagen ECM is modeled with a constitutive law, accounting for alignment and stiffening of the network along directions of tensile strains (Supplementary Note 2). By adopting parameters from our experiments and published cell properties (Supplementary Tables 1 and 2), the model predicts an expanding displacement field around the MF (Fig. 9d). Immediately after seeding, MFs do not contract, with little force transmitted to the collagen substrate (*σ*_*kk*_ = 0); the contraction rate reaches a maximum value ${\dot{ \epsilon }}_{\mathrm{m}}$ in Eq. (1). As MF contraction develops and collagen ECM is deformed and aligned, contraction speed ${\dot{ \epsilon }}_{kk}$ decreases with the increasing stress *σ*_*kk*_ and eventually vanishes after 6 h (Fig. 9e), concomitant with our experimental results (Fig. 5b). The model also predicts that the experimentally shown displacement and strain magnitude decay with distance to the MF (Figs. 1d and 9d).

Next, we integrated a molecular level model to investigate how Mϕ migrate in response to spatiotemporal variation of the deformation fields predicted above (Supplementary Fig. 3). Adhesion molecules (e.g., integrins) act as molecular clutches^26^ (Fig. 9f) that randomly bind and unbind, behaving like a viscous dashpot to resist F-actin retrograde flow (Fig. 9f). For Mϕ directional migration, the binding symmetry between the leading edge pointing to the MF and trailing ends must be broken. Since the speed of stretching at the leading edge is higher than at the rear, the collagen ECM under the Mϕ front end moves faster compared to the rear end. To maintain force equilibrium (Supplementary Note 4), suppression of actin retrograde flow at the leading edge is higher, resulting in higher net protrusion speed compared to the Mϕ rear. Migration velocity relative to ECM velocity is related to the strain rate ${\dot{ \epsilon }}$ as

2 $$V_{{\mathrm{md}}} = \underbrace {\alpha V_{\mathrm{u}}}_{{\mathrm{durotaxis}}} + \underbrace {\alpha R{\dot{ \epsilon }}}_{{\mathrm{strain}}\,{\mathrm{rate}}}.$$

*V*_u_ and *R* represent the retrograde actin flow speed and size of the Mϕ, respectively, and *α* is the transmission factor governed by the stiffness difference of the ECM between Mϕ leading and trailing ends (Supplementary Note 4). Thus two main factors principally guide Mϕ directional migration: the substrate stiffness gradient or durotaxis^24^, and the substrate strain rate caused by the MF contraction. Because Mϕ durotaxis is unlikely to be significant (Fig. 4f), Eq. (2) was approximated as $V_{{\mathrm{md}}} \approx \alpha R{\dot{ \epsilon }}$ (Fig. 9g), suggesting an unloading retrograde flow *V*_u_ < 0.1 nm s^−1^ for the Mϕ that is consistent with previous measurements^27^. Notably, *V*_md_ is the directional migration velocity. Mϕ also randomly migrate with a given local velocity, which is superposed with the directional migration to obtain the total normalized velocity measured in the experiments (Fig. 4g). By decoupling directional from normalized velocity (Supplementary Note 4), fitting the experimental data (Supplementary Table 3), and integrating the strain rate calculated from the MF model, the model predicts that the Mϕ migration velocity decreases with distance to the MF (Fig. 9e). This model prediction and that the migration velocity decay with distance becomes less pronounced as the remodeling time increases is consistent with our experimental result (Figs. 4g and 9e). Overall, our multi-scale model and experimental data show that strain rate is the primary driver of directional Mϕ migration rather than a substrate stiffness gradient.

## Discussion

Close spatial and temporal interaction between inflammatory Mϕ and collagen-producing and -contracting MFs is crucial for tissue repair and fibrosis^5,7^. However, how Mϕ and MFs find each other in the tissue remodeling environment is unclear. Our study provides novel evidence that (1) single contractile MFs can attract multiple migratory Mϕ over distances 20–40-times larger than the Mϕ diameter (up to 1300 µm). (2) Long-range Mϕ–MF communication is promoted by transmission of MF forces through fibrillar ECM and can occur independently from chemotaxis. (3) Static structural ECM cues, such as substrate stiffness or aligned collagen, are insufficient to provide directional Mϕ guidance to MFs. (4) Mϕ perceive and follow dynamic changes in the displacement of their local fibrillar collagen substrate above a critical strain velocity (Fig. 9h). (5) Mϕ mechanosensation requires attachment to collagen via α2β1 integrin and stretch-sensitive membrane ion channels.

One precondition for the transmission of far-ranging mechanical signals is the anisotropic character and geometrical arrangement of fibrillar polymer networks^17,28,29^. Collagen, fibrin, or synthetic fibrillar networks amplify local ECM deformations into mechanical signals that can be perceived by fibroblasts, epithelial cells, and cancer cells over distances of several cell diameters^13,16,30–32^. Although mechanical guidance of adherent cells by mechanical signals propagating through fibrillar ECM seems fundamental, the guidance cues vary with cell type, condition, and observation time. Contracting fibroblasts generate structural ECM changes, such as network compaction, fiber alignment, and fibril buckling. Such changes translate into various directional mechanical signals for adherent cells, including gradients of adhesion sites (haptotaxis)^33^, stiffness gradients (durotaxis)^29,34^, and topographies (contact guidance, e.g., by aligned collagen fibrils)^17,35–38^. Contractile and proteolytically active cells can also establish tracks and tunnel-like structures in 3D collagen gels and tissues that facilitate invasion of tumor and immune cells^39,40^. All these cues have been shown to be perceived by Mϕ^11,41^, which adapt their shapes and migratory activity to ECM surface topographies^42,43^, and switch their migration mode from amoeboid to mesenchymal depending on environmental ECM constraints^44^. Mϕ respond stiff substrates by increasing spreading area, migration speed, actin organization and cell stiffening, proliferation rates and, in some studies, activation/polarization^45,46^.

Durotaxis and haptotaxis are unlikely mechanisms for Mϕ to sense MF over long distances in our experiments for various reasons. First, Mϕ were attracted to contracting MFs from as far as 600 µm in 1 h collagen co-cultures. At this remodeling stage and distance to the MF, the ECM under the responding Mϕ was structurally identical with MF-free gels at micron resolution. Second, ECM displacements measured in these remote regions were <5 µm and likely too small to account for strain stiffening, fibril tension increase, and/or establish ligand density gradients in collagen ECM. Third, fibril alignment and condensation toward the MF force center, typically associated with increased local ECM stiffening^47^, did not promote directional migration of Mϕ. Thus our experimental evidences argue strongly against structural ECM cues guiding Mϕ-to-MF attraction.

Instead, dynamic mechanical attraction signals permit Mϕ mechanosensing of MF contractile activities in remote ECM that does not need to undergo structural changes. Fibroblasts and MFs have been shown to exhibit cyclic contractile events on 2D elastic substrates and 3D fibrillar ECM^19,20^ that can provide Mϕ with continued positional information. Consistently, reproducing similar cyclic pulling event with actuated microneedles substituted the contracting MF as a dynamic force source for Mϕ attraction. Directional migration and/or orientation in response to dynamic changes in the ECM have been observed in various cell types. Pulling and pushing the surface of elastic polyacrylamide polymer substrates or collagen gels with actuated microneedles has been shown to guide fibroblast orientation toward or away from the force source^34,48^. Endothelial cells sense their neighbors over distances of ~10 µm by transmitting and perceiving forces on elastic culture substrates^12^. The molecular basis for dynamic mechanosensing is still being investigated but likely includes changes in the conformation and binding kinetics of cell–ECM adhesion structures^49,50^. Force fluctuations within cell–ECM adhesions (cell tugging) have been shown to enable ECM rigidity (gradient) sensing in migrating cells that generate periodic tractional cytoskeletal forces^50–52^. This sensing mechanism involves the engagement and disengagement of a molecular clutch in focal adhesions^49,53,54^ as supported by our computational model of Mϕ migration in response to MF tugging. At homogeneous ECM stiffness, dynamic mechanosensing is enabled when external force fluctuations are perceived at cell attachment sites, i.e., when the tugging activities of other cells are transmitted through the ECM. Engagement of the molecular clutch is one possible reason for the reduced velocities of Mϕ following a mechanical stimulus compared to randomly migrating Mϕ. However, further studies will be required to pursue this hypothesis.

Mechanoresponses to dynamic signals are dependent on signal amplitude, frequency, and rate^50,55^, which potentially explains that Mϕ in our experiments required an average local ECM displacement velocity of ≥4 µm per 5 min to display a mechanoresponse to remote MF tugging. Strain rate dependence also explains the enhanced attraction of Mϕ in a perimeter of ~250 µm around the MF where ECM displacements were maximal, which was also predicted by our computational model. The question remains why Mϕ attraction to MF decreases with increasing ECM remodeling. We propose that highly organized ECM resists the tractional forces produced by MFs and prevents high ECM displacement amplitudes in the remote gel when tensional homeostasis is reached. Tensional homeostasis is supported by the measured decrease in ECM displacement (∆*d*) amplitudes over time of MF remodeling and the fact that acute stimulation of MF contraction with LPA can induce further ECM displacement even in seemingly static collagen gels. The fact that Mϕ attraction to MFs stalls when tensional homeostasis is reached has the potential for a physiological mechanism terminating Mϕ recruitment when tissue (collagen) organization reaches a level of high resistance to internal (MF) forces and external mechanical challenge.

While chemical signals appear to be crucial for Mϕ–tumor cell interactions^8^, our in vitro results support that chemotaxis is dispensable for Mϕ-to-MF attraction. First, Mϕ efficiently migrated toward MFs even if possible chemotactic gradients were eliminated by slow fluid flow. Second, Mϕ were attracted to actuated microneedles with no cytokine source present. Third, Mϕ were not attracted to MFs co-cultured on pre-remodeled collagen gels or non-deformable collagen-coated culture surfaces. However, mechanical attractions and reported paracrine signaling/chemotaxis between Mϕ and MFs are not mutually exclusive. While chemotactic gradients are comparably short-ranged^15^, local deformations in fibrillar collagen have far-reaching impact on the ECM network^14,16,56^. It is conceivable that dynamic mechanical signals promote long-range attraction, whereas chemotaxis and structural ECM changes are involved in short-range homing of Mϕ to MFs.

By revealing a mechanism of intercellular Mϕ–MF communication through the ECM, our study can inform new strategies to prevent or delay progression of disease conditions associated with tissue remodelling such as cancer and fibrosis^5^. Depletion of Mϕ early after experimentally triggering inflammation in the mouse lungs was shown to slow tissue resolution and result in non-healing wounds; conversely, depleting Mϕ in later stages of tissue remodeling suppresses fibrosis^2,57^. All these studies support that defective Mϕ recruitment blunts MF activation, a desired result in anti-fibrosis treatments. One outcome of our experiments is that inhibition of stretch-activated ion channels and collagen receptors eliminated Mϕ mechanosensing and migration. Specific mechanosensitive membrane channels^58,59^ and fibrillar collagen receptors thus represent potential molecular targets for anti-fibrotic treatments. While the nature of the stretch-activated ion channel(s) remain(s) to be investigated, we here identified α2β1 integrin as the main fibrillar collagen I receptor of in vitro polarized Mϕ1. Integrin α2β1 has been shown to be expressed in tissue Mϕ^60^, as well as integrin α1β1^61^, DDR1^62^, and DDR2^63^. Analyzing collagen receptor expression by Mϕ in different animal models of fibrosis and in human tissue will address this limitation of our in vitro study.

## Methods

### Cells, cell culture, and drugs

Isolation of primary bone marrow-derived Mϕ and lung fibroblasts have been approved by the Animal care committee of Department of Comparative Medicine at University of Toronto, protocol no. 20011598. Lung fibroblasts were explanted from 5–7-week-old C57BL/6 mice (Charles River Laboratories). In brief, mouse lungs were excised, and the tissue were passed through a 70-µm nylon mesh. The cell suspension was then collected in a conical tube and centrifuged 5 min at 150 × *g*. The pellet was resuspended in Dulbecco’s modified Eagle’s medium (DMEM; Life Technologies), supplemented with 10% fetal bovine serum (FBS; Sigma-Aldrich), and penicillin/streptomycin (Wisent, St. Jean-Baptiste, QC, Canada). Primary Mϕ were obtained by flushing femur and tibia of mice and cultured for 7 days in Mϕ medium containing 45% DMEM and F-12, 3% L-glutamine at 200 mM, 10% FBS, 1% penicillin/streptomycin and 20 ng ml^−1^ Mϕ colony-stimulating factor (Life Technologies, Burlington, ON, Canada). The Mϕ were then induced for 2 more days with lipopolysaccharide (100 ng ml^−1^) (Sigma-Aldrich, St. Louis, MO, USA) to induce polarization/activation. For inhibition studies, the ion channel blocker GsMTx-4 (Alomon Laboratories, Jerusalem, Israel) was used. LPA (Sigma-Aldrich) was used to enhance MF contraction in selected experiments.

### Collagen gels

Collagen gels of 250-µm thickness were produced on glass coverslips using pepsin-treated, bovine dermal type I collagen (6.0 mg ml^−1^ type I collagen; Advanced BioMatrix, San Diego, CA). Collagen solutions were neutralized with 0.1 M NaOH to pH 7.4 and diluted to a final concentration of 2 mg ml^−1^. To provide markers at the surface of collagens for the measurement of ECM displacement, collagen solutions were mixed with ferromagnetic micro-beads (2 µm diameter, Bangs Beads, Fishers, IN, 1:10,000 w/v). Glass coverslips were functionalized to firmly attach collagen gels by sequential treatment with (1) 2% aminopropyltriethoxysilane (Sigma A36648; Oakville, ON, Canada) for 15 min, (2) washing in autoclaved water for 5 min, (3) air-drying for 15 min, (4) 0.1% w/w glutaraldehyde (Sigma G5882) for 15 min, and (5) rinsing three times 5 min with autoclaved water at room temperature. The collagen solution was then poured on the center of glass-bottom culture dishes (MatTek, Ashland, MA, USA) and the functionalized coverslips were placed on top. This step ensured the settling of ferromagnetic beads with greater mass toward the bottom surface of the gel prior to polymerization. Glass coverslips and samples were incubated at 37 °C in 5% CO_2_ until polymerization was complete (90–120 min). The coverslips were then gently detached from the glass-bottom dishes by addition of pre-warmed 1× PBS and the coverslips were inverted and stored in 1× PBS. For select experiments, neutralized collagen solution was mixed with Mϕ and MFs in 10:1 ratio and dispensed in thickness of 20 µm onto already polymerized 250-µm-thick collagen gels. During polymerization, cells gravitated to the interphase of the collagen sandwich, which allowed imaging of Mϕ in true 3D ECM within a defined plane of focus (~5–10 µm).

### Analysis of ECM displacement fields

MF were seeded sparsely (2 cells cm^−2^) onto the surface of the collagen gels containing surface bead position markers. Images were acquired at a frame rate of 5 min using inverted phase-contrast microscopy (Zeiss Axiovert 135, Zeiss, Oberkochem, Germany) for 8 h in environmentally controlled conditions (37 °C/5% CO_2_). The initial bead position was determined 1 h after seeding and adhesion of MFs to collagen ECM. Image stacks were aligned using Image J plugin Linear Stack Alignment with scale-invariant feature transform (https://imagej.net/Linear_Stack_Alignment_with_SIFT). All images were then compared one-by-one to the first image using the PIV plugin in ImageJ (https://sites.google.com/site/qingzongtseng/piv#tuto) to map bead displacement over time^64^. To produce heat maps where every vector was displayed at its *x*/*y* position with color-coded magnitude, we used an in-house MATLAB (Mathwork, MA, USA) code. The vectors obtained from PIV were divided into 12 equal sectors of 30 degrees and each sector was divided into increments of 50-µm distances from the centroid of the contractile MF (center of image) up to 1500 µm, and further into rings with 50-µm increments (Fig. 1e). This procedure allowed to calculate the deformation magnitude (sum of all the vectors’ magnitudes) and average displacement (average of all the vectors’ magnitudes) as a function of relative position to the force center (Fig. 1f).

### Live videomicroscopy and analysis of Mϕ migration

MFs were co-cultured with Mϕ on the surface of collagen gels. Mϕ were detached by Accutase incubation (Life Technologies, Burlington, ON, Canada) for 20 min and seeded onto MF-populated collagen gels at different times of establishment of MF deformation field. Samples were placed onto a motorized and environmentally controlled stage and observed using a Zeiss Axiovert 135 inverted phase-contrast microscope with ×5 objective. To exclude contribution of chemotaxis, MF–Mϕ cocultures on collagen were placed in a Chamlide CMB magnetic chamber (Quorum Technologies, Puslinch, ON, Canada). Slow fluid flow (shear force of 0.5 N m^−2^) was applied using a syringe pump (NE-1000, New Era Pump Systems, New York, USA), to pass pre-warmed growth medium over the chamber throughout the observation time.

Mϕ were tracked using series of phase-contrast images using Image J plugin MTrackJ (http://imagej.net/MTrackJ) and trajectories were plotted with respect to the MF (origin of the coordinate system). Mϕ migration velocity (*v*) was calculated by dividing distance (*d*) traveled by the chosen time frame (*t*). In every experiment, Mϕ velocity was related to the displacement of marker beads in their immediate vicinity. In all subsequent experiments, Mϕ displacement was corrected for ECM dragging (Mϕ displacement minus adjacent bead displacement) to only account for Mϕ attraction by active migration by subtracting the velocity of bead movement from Mϕ displacement. Mϕ migration directedness (*D*) and total migration angle (*θ*) were used to classify Mϕ migration trajectories of Mϕ into responsive (0.3 > *D* ≤ 0.5 and *θ* > 90°), attracted (*D* > 0.5 and *θ* < 90°), and non-responsive (*D* < 0.3 or *θ* ≥ 90°). The total migration angle *θ* was defined as the angle between the line connecting the starting point of the Mϕ trajectory and the fibroblast centroid (shortest distance to cell centroid) and the line connecting the starting and end point of the Mϕ trajectory (travel distance). Directedness *D* was defined as a Euclidean length (net displacement) divided by total path length of Mϕ migration tracks.

### Micromanipulation of collagen matrices

Local mechanical loads were applied to collagen gels using microneedle aspiration that mimic dynamic pulling events of MFs for Mϕ. Borosilicate glass capillaries (Harvard Apparatus, Edenbridge, Kent) were pulled using Brown–Flaming micro-pipette puller (Sutter Instrument, Novata, CA) to produce tips of 5-µm diameter. Needles were inserted 5 µm deep into the surface of collagen ECM and negative pressure was applied using a syringe pump. Once the aspirated collagen created a tight seal with the tip, suction was suspended for 15 min during which Mϕ were added. Immobility of non-attached Mϕ and fluorescent particles added to the medium controlled for the absence of any measurable flow. The automated aspiration protocol was chosen to deform the collagen gel at a rate of 1 µm min^−1^, mimicking the kinetics of lateral collagen displacement induced by MFs^19^. Migration of Mϕ and surface marker bead displacement bead was tracked and analyzed as described above.

### Immunofluorescence and confocal reflection microscopy

For immunofluorescence, tissues and 3% paraformaldehyde-fixed and 0.2% Triton X-100-permeabilized cells were incubated with primary antibodies directed against F4/80 (rat IgG2b, BioLegend, CA, USA), followed by incubation with secondary antibodies goat anti-rat Alexa Fluor 488 (Life Technologies, A-11006), Phalloidin-Alexa 568 (Invitrogen, dilution 1:100, cat# A-12379) to stain F-actin (BioLegend, dilution 1:100, cat# 122602), and 4,6-diamidino-2-phenylindole dihydrochloride (DAPI; Sigma-Aldrich, dilution 1:50, D9542) to stain DNA. Fluorescence microscopic images were acquired with an Axio Imager upright microscope equipped with an AxioCam HRm camera, ZEN software (Zeiss), water immersion W N-Achroplan (×10, NA 0.3), and water immersion W Plan-Apochromat (×40, NA 1). The images were then analyzed using ImageJ for the number of Mϕ within a 400-μm circular region of interest around the MFs.

Collagen fibers were visualized using confocal reflectance microscopy using a Leica TCS confocal microscope (Leica TCS SP8, Mannheim, Germany). The images were taken with ×20 objective lens in 5 channels and 40 frames with *z* steps of 1 µm and ×1.3 zoom. To determine collagen compaction, collagen fibril intensities in defined areas around the cell extensions were measured in each sample and normalized against collagen intensities in samples without cells. Local collagen fiber alignment was quantified around fibroblasts using Fast Fourier Transform and Oval Profile (ImageJ plug-in) as described previously^65^. The alignment index was defined based on higher pixel intensities in a specific angle, which was related to the orientation of collagen fibers in the corresponding direction and was quantified by calculation of area under the intensity curve within ±10 degrees of the peak. Figures were assembled in Adobe Photoshop CC; schemes were produced using Adobe Illustrator CC (Adobe Systems, San Jose, CA).

### Flow cytometry

Mϕ were gently detached using Accutase (StemPro, Life Technologies), Fc receptors were blocked using CD16/CD32 antibody, and cells were stained using viability dye eFluor506. Cells were live-labeled with fluorescein isothiocyanate (FITC)-conjugated primary antibody against α2β1 integrin (CD49b, Ha 1/29, Cat# 561891, dilution 1:100) or unconjugated anti-active integrin β1 (CD29, clone 9EG7, Cat# 550531, dilution 1:100) followed by FITC goat anti rat secondary antibody for 60 min. The samples were then run through CytoFlex (Beckman Coulter, Mississauga, ON, Canada), gated for live single cells, and analyzed using the FlowJo software (Treestar, Ashland, OR, USA). All antibodies and reagents for flow cytometry were obtained from BD biosciences (NJ, USA).

### Gene transcription quantification

mRNA was extracted by the PureLink Mini RNA Kit (Invitrogen) according to the manufacturer’s instructions. RNA (500 ng) was reverse-transcribed with the SuperScript VILO cDNA Synthesis Kit (Invitrogen). PCR amplification was performed in triplicate by RT2 SYBR Green ROX (Qiagen, Hilden, Germany) by using StepOnePlus Real-Time PCR System (Applied Biosystems) at 95 °C for 10 min, 40 cycles at 95 °C for 15 s, all at 59 °C annealing temperature for 60 s followed by the melt curve. Relative gene expression levels were calculated by using mouse *Gapdh*, *Hmbs*, and *G6pd* as reference genes. For a list of primers and annealing temperatures, see Table 1.

**Table 1:** Primers for qRT-PCR

Target	Forward 5′→ 3′	Reverse 5′→ 3′
*Itgβ1*	GCGTGGTTGCTGGAATTGTT	GGATTTTCACCCGTGTCCCA
*Itgα1*	TCAGTGGAGAGCAGATCGGA	CACCTTGCCCTGTTCCTCTT
*Itgα2*	CCGGGTGCTACAAAAGTCAT	GTCGGCCACATTGAAAAAGT
*DDR1*	TGGTGGGCCTGGATGATTTC	GTGGGGAAGCTCTGATTGCT
*DDR2*	AGCGAGTCCAGCATGTTCAA	GCGAAGGGGGAAGATTCGAT
*Gapdh*	AGGTCGGTGTGAACGGATTTG	TGTAGACCATGTAGTTGAGGTCA
*Hmbs*	AAGGGCTTTTCTGAGGCACC	AGTTGCCCATCTTTCATCACTG
*G6pd*	CACAGTGGACGACATCCGAAA	AGCTACATAGGAATTACGGGCAA

*qRT-PCR* quantitative reverse transcription polymerase chain reaction

### Statistics

Image analysis was done using ImageJ. Statistical analyses of local angel changes, migration velocity, and image intensity analysis were performed using Microsoft Excel add ins Analysis toolbox and slopestest. All continuous variables (migration velocities and angels) are presented as median ± value range and/or as medians (center lines) of box (25th to 75th percentiles) and whisker (minimum to maximum) plots. The deformation field-reported values are additionally shown as averages. Statistical significance of differences was tested using Student’s *t* test or analysis of variance, followed by post hoc Tukey’s multiple comparison test. Grubb’s test was used to determine and eliminate outliers.

### Reporting summary

Further information on experimental design is available in the Nature Research Reporting Summary linked to this article.

## Supplementary information

Supplementary Information

Description of Additional Supplementary Files

Supplementary Movie 1

Supplementary Movie 2

Supplementary Movie 3

Supplementary Movie 4

Supplementary Movie 5

Reporting Summary

## Data Availability

The data and Matlab codes generated during and/or analyzed during the current study are available from the corresponding authors on reasonable request.
