# Author Correction: Dynamic fibroblast contractions attract remote macrophages in fibrillar collagen matrix

**DOI:** 10.1038/s41467-019-10344-4

**Published:** 2019-05-20

**Authors:** Pardis Pakshir, Moien Alizadehgiashi, Boaz Wong, Nuno Miranda Coelho, Xingyu Chen, Ze Gong, Vivek B. Shenoy, Christopher A. McCulloch, Boris Hinz

**Affiliations:** 1 Laboratory of Tissue Repair and Regeneration, Faculty of Dentistry, University of Toronto, Toronto, ON M5G 1G6 Canada; 2 Faculty of Dentistry, University of Toronto, Toronto, ON M5G 1G6 Canada; 3 Institute of Biomaterials and Biomedical Engineering, University of Toronto, Toronto, ON M5S 3G9 Canada; 4 Department of Chemistry, University of Toronto, Toronto, ON M5S 3E5 Canada; 5 Department of Physiology, University of Western Ontario, London, ON Canada; 6 Center for Engineering Mechanobiology and Department of Materials Science and Engineering, School of Engineering and Applied Science, University of Pennsylvania, Philadelphia, PA 19104 USA

**Keywords:** Motility, Cell migration

Correction to: *Nature Communications* 10.1038/s41467-019-09709-6, published online 23 April 2019.

The original version of this Article contained an error in the spelling of the author Christopher A. McCulloch, which was incorrectly given as Christopher McCulloch. This has now been corrected in both the PDF and HTML versions of the Article.

