# Supplementary material for: Dynamic fibroblast contractions attract remote macrophages in fibrillar collagen matrix
Source: Nat Commun. 2019 Apr 23;10:1850. doi: 10.1038/s41467-019-09709-6 (PMC6478854; doi:10.1038/s41467-019-09709-6)
Supplement: Supplementary file 1 — Supplementary Information [file 41467_2019_9709_MOESM1_ESM.pdf]

Supplementary Material

DYNAMIC FIBROBLAST CONTRACTIONS
ATTRACT REMOTE MACROPHAGES IN FIBRILLAR COLLAGEN MATRIX

Pakshir et al.

Supplementary Notes

Note 1. Chemo-mechanical feedback model for MF contraction

Following our previous work[1], the MF is treated using a combination of passive mechanical and active chemo-mechanical components (Fig. 9a, Supplementary Table 1). The total free energy U_{total} of the MF associated with the elastic strain, myosin recruitment and the mechanosensitive feedback mediated by the adhesions can be written as follows:

$$U_{total} = \frac{K}{2}(\epsilon_{kk})^2 + \mu\tilde{\epsilon}_{ij}\tilde{\epsilon}_{ij} - \frac{1}{3}\int_0^{\epsilon_{kk}} \sigma_{kk} d\epsilon_{ll} - \int_0^{\tilde{\epsilon}_{ij}} \tilde{\sigma}_{ij} d\tilde{\epsilon}_{ij}$$

$$+\frac{\beta}{6}(\rho_{kk} - 3\rho_0)^2 + \frac{\beta}{2}\tilde{\rho}_{ij}\tilde{\rho}_{ij} - \frac{1}{3}\int_0^{\rho_{kk}} \alpha_v\sigma_{kk} d\rho_{kk} - \int_0^{\tilde{\rho}_{ij}} \alpha_d\tilde{\sigma}_{ij} d\tilde{\rho}_{ij}$$

$$+\frac{1}{3}\rho_{kk}\epsilon_{ll} + \tilde{\rho}_{ij}\tilde{\epsilon}_{ij} \quad (1)$$

Here ϵ_{kk}, σ_{kk}, ρ_{kk} and $\tilde{\epsilon}_{ij}$, $\tilde{\sigma}_{ij}$, $\tilde{\rho}_{ij}$ are the volumetric and deviatoric components of the strain, stress, and contractility tensors, which can be related to the strain (ϵ_{ij}), stress (σ_{ij}) and contractility (ρ_{ij}) tensors as follows:

$$\epsilon_{ij} = \frac{1}{3}\epsilon_{kk}\delta_{ij} + \tilde{\epsilon}_{ij},$$

$$\sigma_{ij} = \frac{1}{3}\sigma_{kk}\delta_{ij} + \tilde{\sigma}_{ij},$$

$$\rho_{ij} = \frac{1}{3}\rho_{kk}\delta_{ij} + \tilde{\rho}_{ij}. \quad (2)$$

Also, ρ_0 denotes the myosin motor density when the cell is in quiescent stress-free state, K and μ denote the bulk modulus and shear modulus of the cell, β denotes the chemical stiffness, α_v and α_d denote the volumetric and deviatoric chemo-mechanical feedback parameters. The parameters β and α are related to the molecular mechanisms that regulate the engagement of motors and stress-dependent signaling pathways. When α_v, α_d are large and β is small, under the same stress state, more myosin motors can be recruited and become polarized. To study the kinetics of motor recruitment and the dynamics of contraction, the time dependence of the variation of the total free energy can be written as

$$\frac{dU_{total}}{dt} = \frac{\partial U}{\partial\rho_{kk}}\frac{\partial\rho_{kk}}{\partial t} + \frac{\partial U}{\partial\tilde{\rho}_{ij}}\frac{\partial\tilde{\rho}_{ij}}{\partial t} + \frac{\partial U}{\partial\epsilon_{kk}}\frac{\partial\epsilon_{kk}}{\partial t} + \frac{\partial U}{\partial\tilde{\epsilon}_{ij}}\frac{\partial\tilde{\epsilon}_{ij}}{\partial t} \leq 0, \quad (3)$$

By choosing the rates of change of the contractility and strain in the form

$$\frac{\partial \rho_{kk}}{\partial t} = -k_\rho^v \frac{\partial U}{\partial \rho_{kk}} = -k_\rho^v \left(\frac{1}{3}\epsilon_{kk} + \frac{1}{3}\beta(\rho_{kk} - 3\rho_0) - \frac{1}{3}\alpha_V \sigma_{kk}\right),$$

$$\frac{\partial \tilde{\rho}_{ij}}{\partial t} = -k_\rho^d \frac{\partial U}{\partial \tilde{\rho}_{ij}} = -k_\rho^d \left(\tilde{\epsilon}_{ij} + \beta\tilde{\rho}_{ij} - \alpha_d \tilde{\sigma}_{ij}\right),$$

$$\frac{\partial \epsilon_{kk}}{\partial t} = -k_\epsilon^v \frac{\partial U}{\partial \epsilon_{kk}} = -k_\epsilon^v \left(K\epsilon_{kk} + \frac{1}{3}\beta\rho_{kk} - \frac{1}{3}\sigma_{kk}\right),$$

$$\frac{\partial \tilde{\epsilon}_{ij}}{\partial t} = -k_\epsilon^d \frac{\partial U}{\partial \tilde{\epsilon}_{ij}} = -k_\epsilon^d \left(2\mu\tilde{\epsilon}_{ij} + \tilde{\rho}_{ij} - \tilde{\sigma}_{ij}\right), \tag{4}$$

where $k_\rho^v > 0$, $k_\rho^d > 0$, $k_\epsilon^v > 0$, $k_\epsilon^d > 0$ are the kinetic constants that govern the rates of motor recruitment and cell contraction, we ensure that the rate of change of free energy is always negative $\left(\frac{dU_{total}}{dt} \leq 0\right)$, as required by the second law of thermodynamics. This kinetic law reproduces the linear version of the Hill relation[2], when the rate of motor recruitment and polarization is fast, i.e. $k_\rho^v \to \infty$ and $k_\rho^d \to \infty$. In this limit, the stress and contractility tensor can be derived from Supplementary Equation (4):

$$\sigma_{ij} = \left(\frac{\beta\rho_0}{\beta-\alpha_v} + \frac{K\beta-1/3}{\beta-\alpha_v}\epsilon_{kk} + \frac{\beta/K_\epsilon^v}{\beta-\alpha_v}\dot{\epsilon}_{kk}\right)\delta_{ij} + \frac{2\mu\beta-1}{\beta-\alpha_d}\tilde{\epsilon}_{ij} + \frac{\beta/K_\epsilon^d}{\beta-\alpha_d}\dot{\tilde{\epsilon}}_{ij} \tag{5}$$

$$\rho_{ij} = \left(\frac{\beta\rho_0}{\beta-\alpha_v} + \frac{K\alpha_v-1/3}{\beta-\alpha_v}\epsilon_{kk} + \frac{\alpha_v/K_\epsilon^v}{\beta-\alpha_v}\dot{\epsilon}_{kk}\right)\delta_{ij} + \frac{2\mu\alpha_d-1}{\beta-\alpha_d}\tilde{\epsilon}_{ij} + \frac{\alpha_d/K_\epsilon^d}{\beta-\alpha_d}\dot{\tilde{\epsilon}}_{ij} \tag{6}$$

For the volumetric terms (let $i = k$ and $j = k$), Supplementary Equation (5) can be rearranged to

$$\frac{\sigma_{kk}}{\sigma_m} - \frac{\dot{\epsilon}_{kk}}{\dot{\epsilon}_m} = 1 \tag{7}$$

where

$$\sigma_m = \frac{3\beta\rho_0}{\beta-\alpha_v} + \frac{3K\beta-1}{\beta-\alpha_v}\epsilon_{kk} \text{ and } \dot{\epsilon}_m = \left(3\rho_0 + \frac{3K\beta-1}{\beta}\epsilon_{kk}\right)K_\epsilon^v, \tag{8}$$

where σ_m can be identified as the "stall stress" and $\dot{\epsilon}_m$ is the maximum rate of contraction.

Note 2. Fibrous constitutive model for the collagen ECM

We assume that when the fibrous matrix undergoes stretch, there are two families of fibers: the set of the fibers that align with the direction of the maximum principal stretch and the set of the unaligned fibers that display an isotropic mechanical behavior (Supplementary Table 2). To capture the presence of these two distinct families of aligned and unaligned fibers, following our previous work[3], we assume that the overall strain energy of the ECM consists of two contributions:

$$W_b = \frac{\mu_m}{2}(\overline{I_1} - 3) + \frac{K_m}{2}(J-1)^2, \tag{9}$$

$$W_f = \sum_{a=1}^{3} f(\lambda_a). \tag{10}$$

Here, W_b and W_f denote the strain energy density of the unaligned and aligned fibers, respectively, K_m and μ_m denote the small-strain bulk and shear moduli, respectively and λ_1, λ_2 and λ_3 are the three principal stretches. $\overline{I_1}$ and J are defined as

$$J = \det(\boldsymbol{F}), \tag{11}$$

$$\overline{I_1} = (\lambda_1^2 + \lambda_2^2 + \lambda_3^2)/J^{2/3}, \tag{12}$$

where **F** is the deformation gradient tensor. The strain energy function f is defined by

$$\frac{\partial f}{\partial \lambda_a} = \begin{cases} 0, & \lambda_a < \lambda_l \\ \frac{E_f\left(\frac{\lambda_a-\lambda_l}{\lambda_u-\lambda_l}\right)^n(\lambda_a-\lambda_l)}{n+1}, & \lambda_l \le \lambda_a < \lambda_u \\ E_f\left[\frac{\lambda_u-\lambda_l}{n+1} + \frac{(1+\lambda_a-\lambda_u)^{m+1}-1}{m+1}\right], & \lambda_a \ge \lambda_u \end{cases}, \tag{13}$$

where E_f, n and m are material properties characterizing the degree of the stiffening in response to tensile stresses. In this study, we let $\lambda_l = \lambda_c - (\lambda_c - 1)/8$ and $\lambda_u = \lambda_c + (\lambda_c - 1)/8$. λ_c is the critical stretch corresponding to the onset of strain-stiffening. Like the strain energy, the total stress σ_{ij} can be divided into the unaligned fiber response σ_{ij}^b and aligned fiber response σ_{ij}^f,

$$\sigma_{ij} = \sigma_{ij}^b + \sigma_{ij}^f, \tag{14}$$

which can be calculated from the equations

$$\sigma_{ij}^b = K_m(J-1)\delta_{ij} + \frac{\mu_m(B_{ij} - \frac{1}{3}B_{kk}\delta_{ij})}{J^{5/3}}, \tag{15}$$

$$\sigma_{ij}^{f} = \frac{1}{J}\sum_{a=1}^{3}\frac{\partial f}{\partial \lambda_a}\lambda_a n_i^a n_j^a. \tag{16}$$

Here, n^1, n^1, n^3 are the unit vectors along the three principal directions and B is the left Cauchy-Green tensor, defined as

$$B_{ij} = F_{ik}F_{jk}. \tag{17}$$

Note 3. Cell spreading influenced by ECM stiffness

First, we will explore how the cell spreading is influenced by ECM stiffness using a motor clutch model. For adhesions at lamellipodia tip (Supplementary Fig. 3a), the molecular bonds/clutches, connecting the F-actin with the substrate, were assumed to be able to randomly break or re-engage with a dissociation or association rate of r_{off} or r_{on} respectively. Engagement of the clutches leads to slowing down of the retrograde flow allowing the polymerization at the leading edge to push the cell membrane forward resulting in the spreading of the cell. For the single clutch, the clutch is stretched with the rate of V_r in its lifetime $\tau_0 = \frac{1}{r_{off}}$, exhibiting the average clutch force $V_r\tau_0$. If there are N_c clutches where $N_b = \frac{N_c r_{on}}{r_{on}+r_{off}}$ clutches are engaged, the total force generated is:

$$F_f = N_b V_r \tau_0 k_e = \eta V_r. \tag{18}$$

It becoms evident that adhesions behave like viscous dashpot with viscosity $\eta = N_b k_e \tau_0$ to resist the retrograde flow. Here the effective clutch stiffness is written as $k_e = \frac{k_c k_s}{k_c+k_s}$, since it can be represented by the clutch (with stiffness k_c) and substrate springs (with stiffness k_s) in series. The forces exerted by the myosin motors provide the force to pull the actin filaments towards cell center leading to the retrograde flow V_r. According to Hill's relation[1, 4], myosin force is inversely related with retrograde flow velocity:

$$F_m = F_s\left(1 - \frac{V_r}{V_u}\right). \tag{19}$$

Here F_s is the characteristic myosin force, and V_u is the unloading retrograde flow; the protrusion speed is the difference between polymerization speed V_p and retrograde flow V_r:[5, 6]

$$V_s = V_p - V_r. \quad (20)$$

By combining Supplementary Equations (18-20) and force equilibrium $F_m = F_f$, we can get,

$$V_s = V_p - \frac{F_s V_u}{F_s + \eta V_u}. \tag{21}$$

where the adhesive viscosity $\eta = \frac{N_b \tau_0 k_c k_s}{k_c + k_s}$ is influenced by clutch stiffness, bounded number and ECM stiffness. Based on our previous work[7], cell initial spreading speed determines its final cell spreading area. Thus, our results (Supplementary Fig. 3b) show that the stiffness increases cell spreading for soft ECMs ($k_s < k_c$) while saturates cell spreading area when stiffness is very large ($k_s > k_c$). The transition between increase and saturation occurs at around the clutch stiffness k_c, where the effective stiffness $k_e \approx k_c$ for very stiff ECM ($k_s > k_c$). These results agree well with previous experiments[8] that cell spreading area firstly increases then saturates as the stiffness increases.

Note 4. Mechanism for Mϕ directional migration

Based on the cell spreading model in Supplementary Note 3, we now consider how Mϕ respond to the strain rate of the collagen matrix and migrate towards fibroblast. In contrast with the cell spreading which is assumed as symmetric in all directions, the breaking of symmetry between the front and rear ends is necessary for directional migration. We assume that Mϕ moves at a speed V_m and that the velocity of substrate under the nucleus is V_b. Note that substrate velocity difference between the cell two edges is $2R\dot{\epsilon}$, where $2R$ and $\dot{\epsilon}$ are Mϕ length and strain rate respectively (Supplementary Fig. 3c, Supplementary Table T3). For adhesions at the front end, retrograde flow V_r and local substrate velocity (at the front end) $V_b + R\dot{\epsilon}$ are in the opposite directions.

Thus, the focal adhesion and myosin forces are:

$$F_f^+ = \eta^+ (V_r^+ + V_b + R\dot{\epsilon}), \tag{22}$$

$$F_m^+ = F_s \left(1 - \frac{V_r^+ + V_m}{V_u}\right). \tag{23}$$

At the rear end, the retrograde flow V_r and local substrate velocity $V_b - R\dot{\epsilon}$ share the same direction, so we calculated the focal adhesion and myosin forces as:

$$F_f^- = \eta^-(V_r^- - V_b + R\dot{\epsilon}), \tag{24}$$

$$F_m^- = F_s\left(1 - \frac{V_r^- - V_m}{V_u}\right). \tag{25}$$

Note that at the front, the matrix exhibits higher fibril alignment and larger stiffness k_s compared to the rear end. Since the adhesion molecules/clutches and the ECM are connected in series, the interfacial effective stiffness can be written as $k_e = \frac{k_s k_c}{k_s + k_c}$, leading to larger adhesive viscosity at the front end than the rear end ($\eta^+ > \eta^-$). Conditions of mechanical equilibrium or balance of forces yield:

$$F_f^+ = F_f^-, \quad F_m^+ = F_m^-, \ F_f^+ = F_m^+, \ F_f^- = F_m^-. \tag{26}$$

Previous experiments [6] have found that the protrusion speed is the difference between polymerization speed V_p and retrograde flow V_r:

$$V_s = V_p - V_r. \tag{27}$$

By assuming that the polymerization speed V_p is constant on both sides, we can obtain the corrected Mϕ directional migration velocity (migration velocity relative to substrate velocity):

$$V_{md} = \frac{V_s^+ - V_s^-}{2} - V_b = \frac{V_r^- - V_r^+ - 2V_b}{2}. \tag{28}$$

By combining all above equations, the directional migration velocity (Supplementary Fig. 3d) is:

$$V_{md} = \frac{(\eta^+ - \eta^-)V_u}{\eta^+ + \eta^- + 2V_u\eta^+\eta^-/F_s} + \frac{(\eta^+ - \eta^-)R\dot{\epsilon}}{\eta^+ + \eta^- + 2V_u\eta^+\eta^-/F_s} \tag{29}$$

$$V_{md} = \underbrace{\alpha V_u}_{durotaxis} + \underbrace{\alpha R\dot{\epsilon}}_{strain\ rate}. \tag{30}$$

Here $\alpha = \frac{(1-r)}{1+r+2V_u\eta^+/F_s}$ is the transmission factor, where $r = \frac{\eta^-}{\eta^+}$ is the ratio of adhesive viscosity between the rear and the front ends. Since the adhesive viscosity is related to both the stiffness of bound clutches and the substrate stiffness $\eta = \frac{N_b \tau_0 k_c k_s}{k_c + k_s}$ (Supplementary Note 3), the transmission factor α is affected by the difference between the substrate stiffness at the Mϕ front

and the rear ends. If no stiffness difference appears between two ends (i.e., $r = 1, \alpha = 0$), the Mϕ does not directionally migrate ($V_{md} = 0$). According to Supplementary Equation (30), our model predicts that two key factors influence Mϕ migration — stiffness gradient and strain rate. The stiffness gradient leads to Mϕ migration towards stiffer ECMs is also known as durotaxis[9-11]. Experimental results have shown that after 18 h remodeling of ECM where stiffness gradient (collagen fibril alignment) reaches maximum but the strain rate is small and the Mϕ are not attracted by the MF (Fig. 3f). This indicates the stiffness gradient plays a neglectable role compared to strain rate ($R\dot{\epsilon} \gg V_u$). Thus, the retrograde flow should be small $V_u < 0.1\, nm/s$, which is consistent with previous results[12].

It is important to note that the velocity we predicted is the directional migration velocity. In addition to being attracted by the fibroblast contraction, the Mϕ also show random migration as evidenced by the non-directed movement of macrophages that are far from the fibroblasts. The cell migration velocity measured in the experiments, $\vec{V}_n$ is a superposition of the directional migration velocity $\vec{V}_{md}$ and the random migration velocity $\vec{V}_{mr}$, which can be written as:

$$\vec{V}_n = \vec{V}_{md} + \vec{V}_{mr} \, . \qquad (27)$$

To obtain the directional migration velocity (Fig. 3g), the measured velocity, $\vec{V}_n$, should be projected to the shortest line between the Mϕ and the MF centroid, $i.e.$, $|\vec{V}_{md}| = |\vec{V}_n|\cos(\theta)$ (Supplementary Fig.3e). The measured angle θ is shown in Fig. 3h.

After 1 hour of remodeling, for the Mϕ that are near the boundary of substrate (distance larger than 300 μm), the directional migration velocity $|\vec{V}_{md}|$ decays to nearly zero, but they can still migrate randomly with a velocity $|\vec{V}_{mr}| \sim 0.3\, \mu m/min$.Similarly, after 18 hours of remodeling, Mϕ can still randomly migrate, but do not exhibit any directional migration ($\theta \rightarrow 90°, |\vec{V}_{md}| \rightarrow 0$). We have replotted the *directional* migration velocity as a function of distance from MF after different durations of ECM remodeling in (Supplementary Fig. 3f). According to our theory, the substrate strain rate decays with the distance from the MF centroid (Fig. 9e). We also predict that the Mϕ directional migration speed is linearly dependent on the substrate strain rate ($|\vec{V}_{md}| =$

$\alpha R\dot{\epsilon}$). Combining these two predictions, we find that the macrophage directional migration speed should decrease with the distance from the MF centroid. Near the edge of the substrate strain field (distance larger than $300\mu m$), the macrophage directional migration speed is predicted to vanish. Clearly, the experimental measurements plotted in (Supplementary Fig.3f) are consistent with our model predictions. The random migration velocity $|\vec{V}_{mr}| = |\vec{V}_n|\sin(\theta)$ of Mϕ that are far from MF slightly increases with remodeling time. This can be explained by the fact that cells randomly migrate faster on stiffer ECM[13]. In addition, to fit for the directional migration velocity in (Supplementary Fig. 3f), a scale factor $SF \approx 10$ ($\left|\vec{V}_{md}\right| = \alpha R\dot{\epsilon} * SF$) was introduced to account for possible variations of the myosin, clutch numbers and strain rate.

Supplementary Tables

Supplementary Table 1: MF mechanical properties

Fibroblast			
Parameters	Physical meaning	Values	Reference/Origin
ρ_0	Motor density (quiescent state)	1 kPa	[1]
K	Bulk modulus	833 Pa	[1]
μ	Shear modulus	384 Pa	[1]
β	Chemical stiffness	2.77 kPa^{-1}	[1]
α_d	Deviatoric feedback parameter	2.6 kPa^{-1}	[1]
α_v	Volumetric feedback parameter	2.6 kPa^{-1}	[1]
K_ϵ^d	Deviatoric kinetic parameter	$5 \times 10^{-8}\ \mathrm{Pa}^{-1}\mathrm{s}^{-1}$	[4, 14, 15]
K_ϵ^v	Volumetric kinetic parameter	$5 \times 10^{-8}\ \mathrm{Pa}^{-1}\mathrm{s}^{-1}$	[4, 14, 15]
a	Length	200 µm	Measurement
b	Width	60 µm	Measurement
c	Height	60 µm	Measurement

Supplementary Table 2: Mechanical properties of the collagen substrate

Collagen substrate			
Parameters	Physical meaning	Values	Origin
μ_m	Unaligned fiber shear modulus	50.0 Pa	[3]
K_m	Unaligned fiber bulk modulus	33.3 Pa	[3]
E_f	Aligned fiber initial modulus	1000 Pa	[3]
λ_c	Critical stretch for fiber alignment	1.001	[3]
n	Fiber transition parameter	5	[3]
m	Fiber stiffening parameter	10	[3]

Supplementary Table 3: Mϕ migration parameters

Macrophage			
Parameters	Physical meaning	Values	Origin
α	Transmission factor	0.9	Fitting parameter
R	Mϕ radius	10 μm	Measurement
V_u	Unloading retrograde flow	0.01~0.1 nm/s	[6]
$\dot{\epsilon}$	ECM strain rate	$10^{-5}\sim10^{-4}\ s^{-1}$	Simulation results

Supplementary Figures

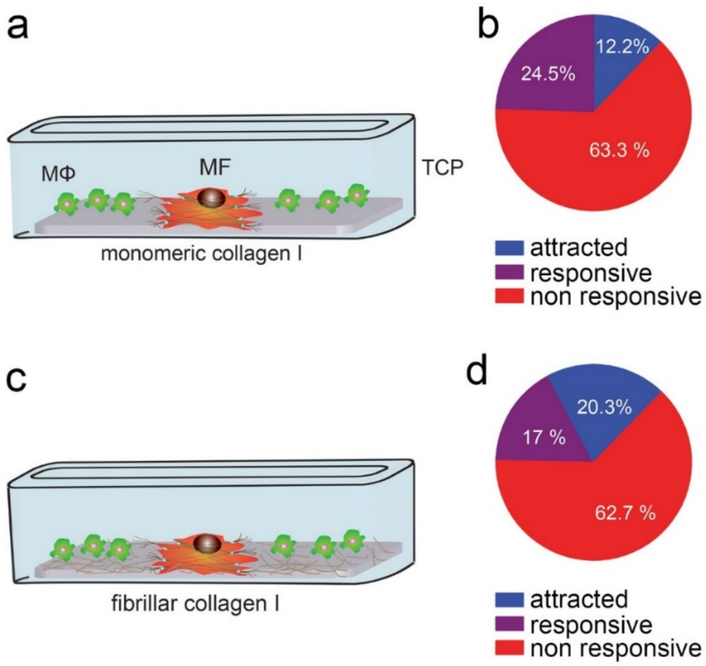

Supplementary Figure 1. *Mϕ are not attracted to MF on non-deformable ECM.* (a) Schematic representation of Mϕ-MF co-cultures on 5 µm thin monomeric collagen type I-coated tissue culture plastic (TCP). (b) Percentages of attracted, responsive, and non-responsive Mϕ in response to MF (69 Mϕ, 10 independent experiments). (c) Schematic representation of Mϕ-MF co-cultures on 5 µm thin fibrillar collagen type I -coated TCP. (d) Percentages of attracted, responsive, and non-responsive Mϕ in response to MF (49 Mϕ, 10 independent experiments).

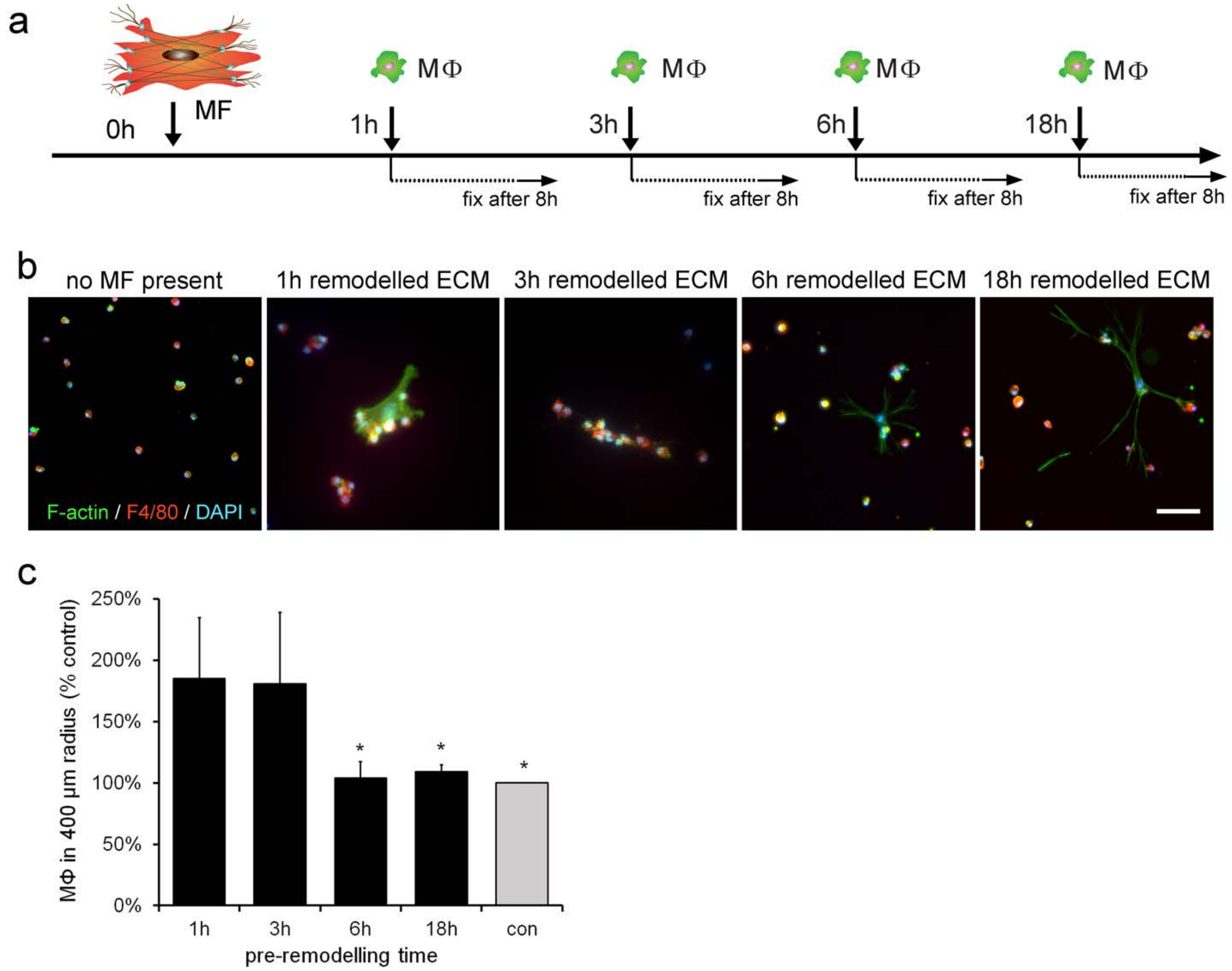

Supplementary Figure 2. ECM pre-remodelling does not result in Mϕ accumulation around MFs.

(a) MFs remodelled collagen ECM for 1-18 h. After the indicated remodelling times, Mϕ were added to the pre-organized ECM in continued presence of MFs and allowed to migrate for another 8 h. (b) Samples were then processed for immunostaining against F4/80 (Mϕ marker, red), F-actin (green) and DAPI (blue). (c) The number of Mϕ in a 400 µm radius around the MFs was automatically determined from F4/80 staining and plotted as a function of ECM pre-remodelling duration. Controls were matrices with no MF present. Shown are averages±SD from three independent experiments (*$p \leq 0.05$ using ANOVA followed by a post-hoc Tukey's multiple comparison test). Scale bar: 100 µm.

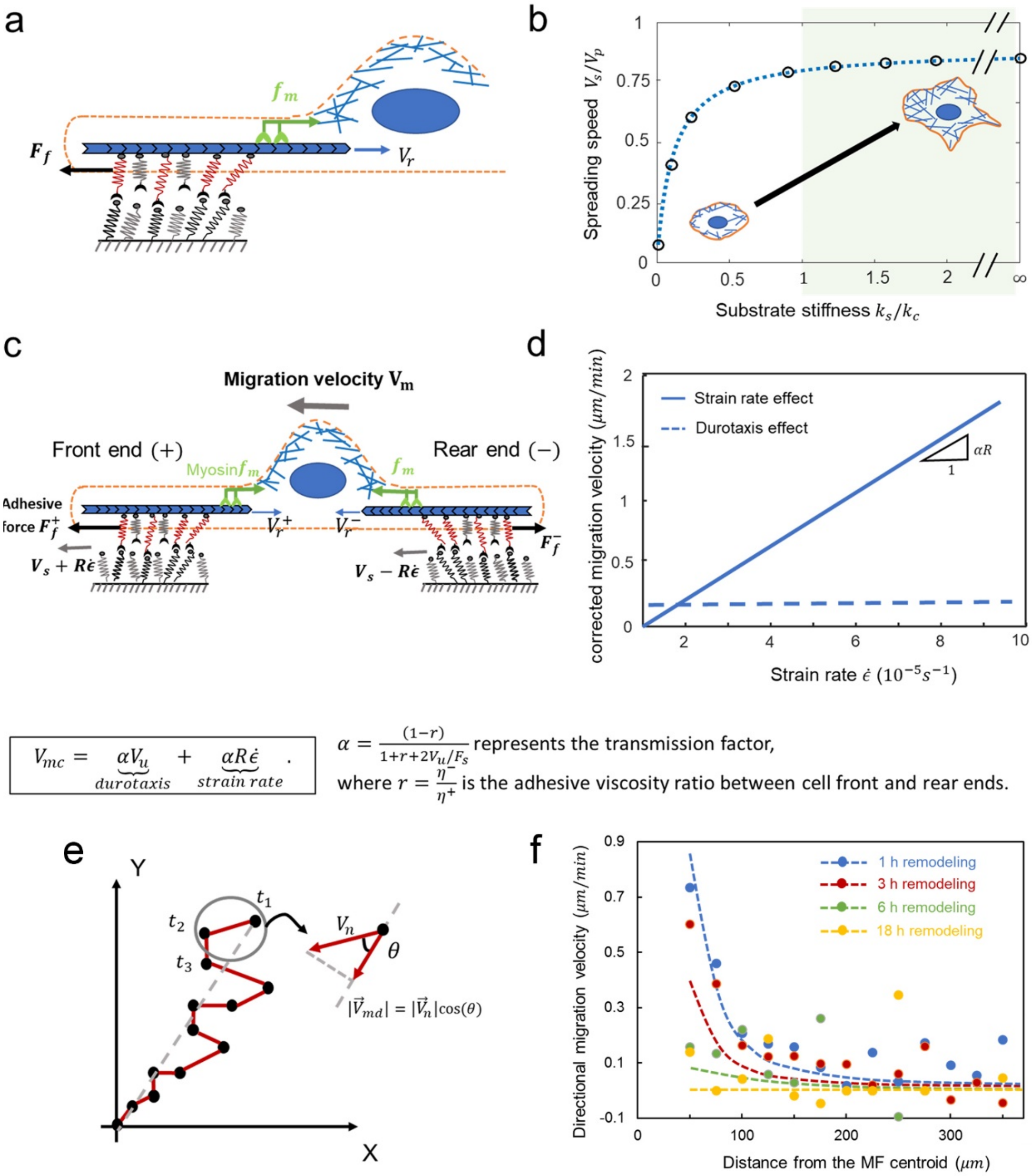

Supplementary Figure 3: Molecular level model of Mϕ migration. The molecular level model predicts the influence of matrix stiffness on cell spreading. (b) The model predicts that the cell spreading first increases and then saturates as the ECM stiffness increases. (c) The molecular level model predicts the migration of Mϕ in response to the substrate deformation. (d) Both, strain rate $\alpha R\dot{\epsilon}$ and durotaxis αV_u (dashed line) influence Mϕ migration speed V_m. (e) Schematic of the migration path of Mϕ. The directional migration velocity $|\vec{V}_{md}|$ should be the projection of normalized migration velocity $|\vec{V}_n|$ on the shortest line between the point where the Mϕ is introduced and the centroid of the MF towards which it moves (grey dashed line). (f) The directional migration velocity plotted as a function of distance from the MF centroid for 1, 3, 6 and 18 hours of remodeling. The dots show the experimental data, and the dashed lines are the predictions from our model.

Supplementary References

1. Shenoy, V.B., Wang, H. & Wang, X. A chemo-mechanical free-energy-based approach to model durotaxis and extracellular stiffness-dependent contraction and polarization of cells. *Interface Focus* **6**, 20150067 (2016).
2. Holmes, J.W. Teaching from classic papers: Hill's model of muscle contraction. *Adv Physiol Educ* **30**, 67-72 (2006).
3. Wang, H., Abhilash, A.S., Chen, C.S., Wells, R.G. & Shenoy, V.B. Long-range force transmission in fibrous matrices enabled by tension-driven alignment of fibers. *Biophys J* **107**, 2592-2603 (2014).
4. Mitrossilis, D. et al. Single-cell response to stiffness exhibits muscle-like behavior. *Proc Natl Acad Sci U S A* **106**, 18243-18248 (2009).
5. Nisenholz, N. et al. Active mechanics and dynamics of cell spreading on elastic substrates. *Soft Matter* **10**, 7234-7246 (2014).
6. Giannone, G. et al. Periodic lamellipodial contractions correlate with rearward actin waves. *Cell* **116**, 431-443 (2004).
7. Gong, Z. et al. Matching material and cellular timescales maximizes cell spreading on viscoelastic substrates. *Proc Natl Acad Sci U S A* **115**, E2686-E2695 (2018).
8. Tee, S.Y., Fu, J., Chen, C.S. & Janmey, P.A. Cell shape and substrate rigidity both regulate cell stiffness. *Biophys J* **100**, L25-27 (2011).
9. Sunyer, R. et al. Collective cell durotaxis emerges from long-range intercellular force transmission. *Science* **353**, 1157-1161 (2016).
10. Plotnikov, S.V., Pasapera, A.M., Sabass, B. & Waterman, C.M. Force fluctuations within focal adhesions mediate ECM-rigidity sensing to guide directed cell migration. *Cell* **151**, 1513-1527 (2012).
11. Discher, D.E., Janmey, P. & Wang, Y.L. Tissue cells feel and respond to the stiffness of their substrate. *Science* **310**, 1139-1143 (2005).
12. Hui, K.L. & Upadhyaya, A. Dynamic microtubules regulate cellular contractility during T-cell activation. *Proc Natl Acad Sci U S A* **114**, E4175-E4183 (2017).
13. Bangasser, B.L. et al. Shifting the optimal stiffness for cell migration. *Nat Commun* **8**, 15313 (2017).
14. Mitrossilis, D. et al. Real-time single-cell response to stiffness. *Proc Natl Acad Sci U S A* **107**, 16518-16523 (2010).
15. Etienne, J. et al. Cells as liquid motors: mechanosensitivity emerges from collective dynamics of actomyosin cortex. *Proc Natl Acad Sci U S A* **112**, 2740-2745 (2015).
