# Supplementary material for: Dynamic fibroblast contractions attract remote macrophages in fibrillar collagen matrix
Source: Nat Commun. 2019 Apr 23;10:1850. doi: 10.1038/s41467-019-09709-6 (PMC6478854; doi:10.1038/s41467-019-09709-6)
Supplement: Supplementary file 2 — Description of Additional Supplementary Files [file 41467_2019_9709_MOESM2_ESM.pdf]

Description of Additional Supplementary Files

File Name: Supplementary Movie 1
Description: *Time lapse videomicroscopy of MF produced deformation field.* Images were taken every hour for 9 h. Video corresponds to example images in Fig. 1d. Single MFs were attached to the top of fibrillar collagen ECM provided with surface marker beads and allowed to remodel the ECM for 3 h. MF-induced deformation fields are analyzed using PIV and displayed as vectors with color coded magnitudes (red-high, blue-low displacement). Scale bar: 100 µm.

File Name: Supplementary Movie 2
Description: *Time lapse videomicroscopy of Mϕ-MF co-cultures on fibrillar collagen ECM.* Mφ were co-cultured with MFs on fibrillar collagen ECM with surface marker beads and video-recorded. Images were taken every 5 min over 9 h; shown are 6 h, 10 min. Mφ (false-colored, green) and beads (false-colored, red) were automatically identified on basis of size, circularity and brightness in phase contrast images and tracked. Video corresponds to example images in Fig 2b. Scale bar: 100 µm.

File Name: Supplementary Movie 3
Description: *Time lapse videomicroscopy of Mϕ-MF co-cultures on fibrillar collagen ECM.* Mφ were co-cultured with MFs on fibrillar collagen ECM with surface marker beads. Images were taken every 5 min over 8 h. Mφ track lengths and velocity were compared with displacement of adjacent microbeads. All Mφ tracks were subsequently corrected for bead displacement (passive dragging). Video corresponds to example images in Fig. 2c. Scale bar: 100µm.

File Name: Supplementary Movie 4
Description: *Time lapse videomicroscopy of Mϕ-MF co-cultures on collagen ECM under slow fluid flow.* Mφ were co-cultured with MFs on fibrillar collagen ECM and a slow fluid flow was applied to skew/eliminate possible chemotactic gradients. The movement of the beads mixed in the medium is shown side by side. Images were taken every 5 min over 6 h. Video corresponds to example images in Fig. 2d. Scale bar: 100µm.

File Name: Supplementary Movie 5
Description: *Time lapse videomicroscopy of Mϕ responding to deformation fields induced by a micromanipulator.* Mφ seeded onto collagen ECM with surface marker beads in the absence of MF with inducing a lateral collagen deformation. Images were taken every 5 min over 8 h. Mφ migration was tracked from phase contrast movies. Video corresponds to example images in Fig. 4a. Scale bar: 100µm.
