# Supplementary material for: Dynamic fibroblast contractions attract remote macrophages in fibrillar collagen matrix
Source: Nat Commun. 2019 Apr 23;10:1850. doi: 10.1038/s41467-019-09709-6 (PMC6478854; doi:10.1038/s41467-019-09709-6)
Supplement: Supplementary file 8 — Reporting Summary [file 41467_2019_9709_MOESM8_ESM.pdf]

nature research

Corresponding author(s): Boris Hinz

Last updated by author(s): Mar 18, 2019

# Reporting Summary

Nature Research wishes to improve the reproducibility of the work that we publish. This form provides structure for consistency and transparency in reporting. For further information on Nature Research policies, see Authors & Referees and the Editorial Policy Checklist.

## Statistics

For all statistical analyses, confirm that the following items are present in the figure legend, table legend, main text, or Methods section.

| n/a | Confirmed | |
|---|---|---|
| ☐ | ☒ | The exact sample size (*n*) for each experimental group/condition, given as a discrete number and unit of measurement |
| ☒ | ☐ | A statement on whether measurements were taken from distinct samples or whether the same sample was measured repeatedly |
| ☐ | ☒ | The statistical test(s) used AND whether they are one- or two-sided<br>*Only common tests should be described solely by name; describe more complex techniques in the Methods section.* |
| ☐ | ☒ | A description of all covariates tested |
| ☒ | ☐ | A description of any assumptions or corrections, such as tests of normality and adjustment for multiple comparisons |
| ☐ | ☒ | A full description of the statistical parameters including central tendency (e.g. means) or other basic estimates (e.g. regression coefficient) AND variation (e.g. standard deviation) or associated estimates of uncertainty (e.g. confidence intervals) |
| ☐ | ☒ | For null hypothesis testing, the test statistic (e.g. *F*, *t*, *r*) with confidence intervals, effect sizes, degrees of freedom and *P* value noted<br>*Give P values as exact values whenever suitable.* |
| ☒ | ☐ | For Bayesian analysis, information on the choice of priors and Markov chain Monte Carlo settings |
| ☒ | ☐ | For hierarchical and complex designs, identification of the appropriate level for tests and full reporting of outcomes |
| ☒ | ☐ | Estimates of effect sizes (e.g. Cohen's *d*, Pearson's *r*), indicating how they were calculated |

*Our web collection on statistics for biologists contains articles on many of the points above.*

## Software and code

Policy information about availability of computer code

| | |
|---|---|
| Data collection | Zeiss microscopy software, Micro-Manager Open Source Microscopy Software |
| Data analysis | Graphpad Prism, Origin, Microsoft Excel, In house Matlab code, Fiji (Image j) |

For manuscripts utilizing custom algorithms or software that are central to the research but not yet described in published literature, software must be made available to editors/reviewers. We strongly encourage code deposition in a community repository (e.g. GitHub). See the Nature Research guidelines for submitting code & software for further information.

## Data

Policy information about availability of data

All manuscripts must include a data availability statement. This statement should provide the following information, where applicable:

- Accession codes, unique identifiers, or web links for publicly available datasets
- A list of figures that have associated raw data
- A description of any restrictions on data availability

The data and Matlab codes generated during and/or analysed during the current study are available from the corresponding authors upon on reasonable request.

# Field-specific reporting

Please select the one below that is the best fit for your research. If you are not sure, read the appropriate sections before making your selection.

☒ Life sciences ☐ Behavioural & social sciences ☐ Ecological, evolutionary & environmental sciences

For a reference copy of the document with all sections, see nature.com/documents/nr-reporting-summary-flat.pdf

# Life sciences study design

All studies must disclose on these points even when the disclosure is negative.

| | |
|---|---|
| Sample size | Statistical test were performed as indicated in Methods and Figure Legends to assure appropriate sample sizes. |
| Data exclusions | No data were excluded except from experiments that failed for obvious technical reasons. |
| Replication | Experiments have been repeated several times by the same or different experimenters with reproducible outcomes. |
| Randomization | This is not relevant for the study. |
| Blinding | Blinding was not relevant for the study. |

# Reporting for specific materials, systems and methods

We require information from authors about some types of materials, experimental systems and methods used in many studies. Here, indicate whether each material, system or method listed is relevant to your study. If you are not sure if a list item applies to your research, read the appropriate section before selecting a response.

## Materials & experimental systems

| n/a | Involved in the study | |
|---|---|---|
| ☐ | ☒ | Antibodies |
| ☐ | ☒ | Eukaryotic cell lines |
| ☒ | ☐ | Palaeontology |
| ☐ | ☒ | Animals and other organisms |
| ☒ | ☐ | Human research participants |
| ☒ | ☐ | Clinical data |

## Methods

| n/a | Involved in the study | |
|---|---|---|
| ☒ | ☐ | ChIP-seq |
| ☐ | ☒ | Flow cytometry |
| ☒ | ☐ | MRI-based neuroimaging |

## Antibodies

| | |
|---|---|
| Antibodies used | IF: Phalloidin-Alexa 568 (Life TechnologiesInvitrogen, dilution 1:100, cat# A-12379) to stain F-actin (Biolegend, dilution 1:100, cat# 122602) and 4,6-Diamidino-2-phenylindole dihydrochloride (DAPI, Sigma-Aldrich, dilution 1:50, D9542) to stain DNA. Flow cytometry: antibody against α2β1 integrin (CD49b, Ha 1/29, Cat# 561891, dilution 1:100) or unconjugated anti-active integrin β1 (CD29, clone 9EG7, cat# 550531, dilution 1:100) |
| Validation | All antibodies have been validated in house in addition to what is provided by the manufacturer. Validation includes use of the antibody on cell types that are negative for the epitope, secondary antibody only controls, IgG controls. |

## Eukaryotic cell lines

Policy information about cell lines

| | |
|---|---|
| Cell line source(s) | Primary bone marrow-derived macrophages and lung fibroblasts |
| Authentication | Lung fibroblasts were explanted from 5-7 weeks-old C57BL/6 mice (Charles River Laboratories). In brief, mouse lungs were excised and the tissue were passed through a 70 µm nylon mesh. The cell suspension was then collected in a conical tube and centrifuged 5 min at 150×g. The pellet was then resuspended in DMEM (Life Technologies), supplemented with 10% fetal bovine serum (Sigma-Aldrich), and penicillin/streptomycin (Wisent, St. Jean-Baptiste, QC, Canada). Primary Mϕ were obtained by flushing femur and tibia of mice and cultured for 7 days in Mϕ medium containing 45% DMEM and F-12, 3% L-glutamine at 200 mM, 10% FBS, 1% penicillin/streptomycin and 20 ng/ml Mϕ colony stimulating factor (M-CSF) |
| Mycoplasma contamination | Primary bone marrow dervied macrophages and lung fibroblasts tested negative for Mycoplasma contamination. |
| Commonly misidentified lines (See ICLAC register) | N/A |

## Animals and other organisms

Policy information about studies involving animals; ARRIVE guidelines recommended for reporting animal research

| | |
|---|---|
| Laboratory animals | 5-7 weeks-old C57BL/6 mice |

| | |
|---|---|
| Wild animals | N/A |
| Field-collected samples | N/A |
| Ethics oversight | N/A |

Note that full information on the approval of the study protocol must also be provided in the manuscript.

# Flow Cytometry

## Plots

Confirm that:

☒ The axis labels state the marker and fluorochrome used (e.g. CD4-FITC).

☒ The axis scales are clearly visible. Include numbers along axes only for bottom left plot of group (a 'group' is an analysis of identical markers).

☐ All plots are contour plots with outliers or pseudocolor plots.

☒ A numerical value for number of cells or percentage (with statistics) is provided.

## Methodology

| | |
|---|---|
| Sample preparation | Macrophages were detached using Accutase, Fc receptors were blocked using CD16/CD32 antibody and stained using viability dye eFlouor506. Cells were live-labeled with FITC-conjugated primary antibody against α2β1 integrin or unconjugated anti-active integrin β1 followed by FITC goat anti rat secondary antibody for 60 min. |
| Instrument | CytoFlex (Beckman Coulter) |
| Software | CytoFlex sofware. The post analysis and gating was done on FlowJo software. |
| Cell population abundance | Polarity of bone marrow derived macrophages were throughly investigated by flow cytometry in our most recent publication: Lodyga, M. et al. Cadherin-11–mediated adhesion of macrophages to myofibroblasts establishes a profibrotic niche of active TGF-β (Journal Cover Page). Science Signalling (2019) |
| Gating strategy | Forward versus side scatter (FSC vs SSC) gating was used to identify cells of interest based on size and granularity (complexity). Macrophages were gated for live cells. A forward scatter height (FSC-H) vs. forward scatter area (FSC-A) was used to identify doublets. The live cells were then gated for single cells. Single parameter histograms were then used to identify b1 or a2 positive macrophages from the single and live population. |

☒ Tick this box to confirm that a figure exemplifying the gating strategy is provided in the Supplementary Information.
